# Altered hippocampal neurogenesis in a mouse model of autism revealed by genetic polymorphisms and by atypical development of newborn neurons

**DOI:** 10.1038/s41598-024-53614-y

**Published:** 2024-02-26

**Authors:** Isabel Barón-Mendoza, Montserrat Mejía-Hernández, Karina Hernández-Mercado, Jessica Guzmán-Condado, Angélica Zepeda, Aliesha González-Arenas

**Affiliations:** Departamento de Medicina Genómica y Toxicología Ambiental, Instituto de Investigaciones Biomédicas, Universidad Nacional Autónoma de México, 04510 Mexico City, México

**Keywords:** Adult neurogenesis, Autism spectrum disorders

## Abstract

Individuals with autism spectrum disorder (ASD) often exhibit atypical hippocampal anatomy and connectivity throughout their lifespan, potentially linked to alterations in the neurogenic process within the hippocampus. In this study, we performed an in-silico analysis to identify single-nucleotide polymorphisms (SNPs) in genes relevant to adult neurogenesis in the C58/J model of idiopathic autism. We found coding non-synonymous (Cn) SNPs in 33 genes involved in the adult neurogenic process, as well as in 142 genes associated with the signature genetic profile of neural stem cells (NSC) and neural progenitors. Based on the potential alterations in adult neurogenesis predicted by the in-silico analysis, we evaluated the number and distribution of newborn neurons in the dentate gyrus (DG) of young adult C58/J mice. We found a reduced number of newborn cells in the whole DG, a higher proportion of early neuroblasts in the subgranular layer (SGZ), and a lower proportion of neuroblasts with morphological maturation signs in the granule cell layer (GCL) of the DG compared to C57BL/6J mice. The observed changes may be associated with a delay in the maturation trajectory of newborn neurons in the C58/J strain, linked to the Cn SNPs in genes involved in adult hippocampal neurogenesis.

## Introduction

Autism spectrum disorder (ASD) comprises a group of neurodevelopmental disorders characterized by persistent deficits in social interaction/communication and the presence of repetitive and stereotyped behaviors^1^. It is estimated that 1 in 100 children worldwide has ASD^2^. The etiology of autism is polygenic and, in most cases, it is idiopathic^3^.

Interestingly, dysregulation in the neurogenic process has been proposed as one of the underlying mechanisms associated with changes in brain structure in ASD^4^. There is evidence of aberrant cortical lamination in children with ASD, which may arise from disturbances in cell proliferation, migration, and differentiation during prenatal development^5^. Moreover, toddlers with autism exhibit brain overgrowth during the first years of life, which may result from altered cell proliferation^4^. In line with this, in vitro experiments using induced pluripotent stem cells (iPSCs)-derived neural progenitors from individuals with autism with increased brain volume have demonstrated accelerated proliferation^6^, decreased proliferation when derived from individuals who do not have macrocephaly^7^, and impairments during the determination of neuronal subtype^8^. There is also strong evidence that genes with autism-associated variants are involved in the neurogenic regulation^3,4,9^.

Adult neurogenesis in humans is still a topic of debate; however, in mice, the subgranular zone (SGZ) of the hippocampal dentate gyrus (DG) is one of the well-recognized adult neurogenic niches^10,11^. Adult neurogenesis in rodents plays a crucial role in hippocampal functionality and plasticity^12^, while individuals with autism often display atypical hippocampal anatomy and connectivity throughout their lifespan^13^, as well as changes in the volume and cytoarchitecture of the DG, particularly in the granule cell layer (GCL)^14,15^. Hence, the evaluation of the neurogenic process in the hippocampus of autism models is a relevant topic for understanding the potential contribution of this process in ASD.

Adult neurogenesis in the mouse DG involves a series of tightly regulated cellular events that allow progenitor cells to differentiate and develop into fully functional mature granule cells (GC)^16–18^. Type 1 neural stem cells (NSC), also known as radial glial-like progenitors, express stem markers nestin and GFAP (glial fibrillary acidic protein). Upon specific signaling, NSC residing in the SGZ of the DG exit their quiescent state and start dividing, giving rise to intermediate progenitor cells (Type 2a cells) which exhibit high proliferative activity and a non-radial glial-like morphology, while still expressing glial markers. Type 2a cells generate intermediate progenitor cells (Type 2b cells) that express early neuronal specification markers such as DCX (Doublecortin) and PSA-NCAM (Polysialylated-neural cell adhesion molecule)^16,19^. Type 2 cells that survive the critical period divide and differentiate into neuroblasts (Type 3 cells)^20^, which express DCX and PSA-NCAM and exhibit varying morphologies depending on their maturation stage. In early stages, they may display short, plump dendritic processes or none. In the final stage of morphological maturation, fully differentiated young neurons develop a long apical dendrite with branches reaching the molecular layer, parallel to the expression of calretinin^21^. Only a small subset of the newborn cells matures into calbindin-expressing granule cells, which are then available to integrate into pre-established DG circuits^16,19^.

The adult neurogenic process is also controlled by coordinated expression patterns of several genes^17,22,23^. Interestingly, common genetic variants in inbred mice are associated with strain differences in adult hippocampal neurogenesis, suggesting a strong contribution of polymorphisms in the regulation of the neurogenic process^23^.

Our group has previously identified single-nucleotide polymorphisms (SNPs) in genes essential for brain development and plasticity in the C58/J murine model of idiopathic ASD^24^. The C58/J strain exhibits alterations in hippocampal cytoarchitecture and hippocampus-dependent learning deficits^24–27^, which may be linked, among other factors, to impairments in hippocampal neurogenesis.

Given the impact that specific genetic alterations may have on the neurogenic process and the potential dysregulation of neurogenesis in ASD after birth, we conducted an in-silico study of single-nucleotide polymorphisms (SNPs) in genes relevant to adult neurogenesis in the C58/J model and analyzed whether the hippocampal neurogenic process in this strain could potentially exhibit alterations based on the predicted changes.

## Results

### The C58/J strain displays non-synonymous SNPs in genes associated with the regulation of adult neurogenesis

According to in vivo and in vitro experimental data curated and reported by the MANGO database, a total of 397 genes are expressed during the stages of proliferation, differentiation, survival, migration, dendritogenesis, and maturation in adult neurogenesis^17^. These genes are also expressed in specific cell stages defined by MANGO as precursor cells, stem cells (Type 1), undetermined progenitors (Type 2a), determined progenitors (Type 2b), neuroblast-like cells (Type 3), new neurons, immature and mature neurons, and doublecortin (DCX)-positive cells^17^ (See supplementary Table S1 for MANGO criteria and definitions).

**Figure 1:**
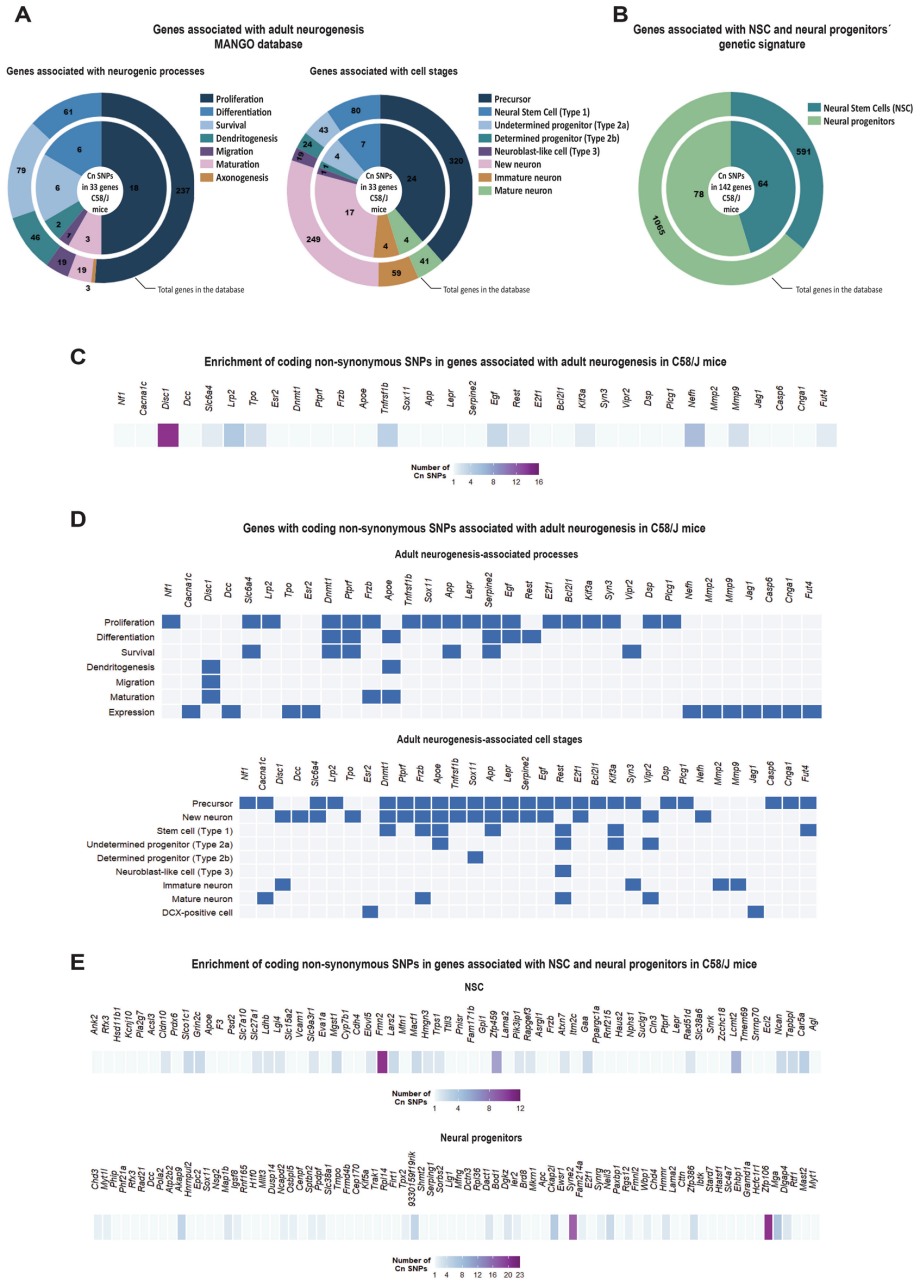
**Neurogenesis-associated genes with coding non-synonymous SNPs in the C58/J strain**. In silico analysis of coding non-synonymous (Cn) single-nucleotide polymorphisms (SNPs) in genes relevant to adult neurogenesis in the mouse DG. (**A**) Among the 397 genes associated with processes (*left*) and cell stages (*right*) involved in adult neurogenesis, according to the MANGO database (*data inside the large circle*), 33 genes carried at least one Cn SNP in C58/J mice (*data inside the small circle*). (**B**) Among the genes characterizing the genetic profile of neural stem cells (NSC) (591 genes) and neural progenitors (1065 genes) in the mouse DG, as reported by Artegiani (2017) (*data inside the large circle*), 142 genes carried at least one Cn SNP in C58/J mice, with 64 genes corresponding to NSC and 78 genes to the neural progenitors (*data inside the small circle*). (**C**) The 33 genes reported by the MANGO database display from 1 to 16 Cn SNPs in C58/J mice in comparison with the control C57BL/6 J strain. (**D**) The genes with Cn SNPs in C58/J mice are expressed during specific neurogenic processes (*upper panel*: proliferation, differentiation, survival, dendritogenesis, migration, maturation) and throughout different cell stages (*inferior panel*: stem cells [Type 1], undetermined progenitors [Type 2a], determined progenitors [Type 2b], neuroblast-like cells [Type 3], immature neurons, mature neurons, DCX-positive cells), according to the MANGO database. (**E**) 64 and 78 genes corresponding to the NSC (*upper panel*) and neural progenitors’ (*inferior panel*) datasets, respectively, display from 1 to 23 Cn SNPs in C58/J mice.

Thus, to predict potential changes in adult neurogenesis in the C58/J model of autism, we conducted an in-silico analysis of the 397 genes reported by MANGO to identify single-nucleotide polymorphisms (SNPs) in the C58/J strain, compared to the control C57BL/6 J strain (WT). We used the Sanger4 Dataset available in the Mouse Phenome Database (MPD)^28–30^. Our analysis revealed that among the 397 genes, 33 of them carried at least one non-synonymous SNP in coding regions (Cn SNPs), leading to changes in the coding amino acid sequence, in the C58/J strain. Notably, the *Disc1* gene showed the highest enrichment of coding non-synonymous (Cn) SNPs (Fig. 1A,C, Supplementary Table S2). Additionally, within the Cn SNPs of the *Disc1* gene, three (rs31943453, rs31944226, rs215748054) were identified as highly prone to damaging the protein structure based on impact predictions conducted using the PolyPhen-2 platform^31^ (Supplementary Table S3).

According to MANGO, among the 33 genes with Cn SNPs in C58/J mice, 18 are involved in proliferation (*Nf1, Slc6a4, Lrp2, Dnmt1, Ptprf, Frzb, Tnfrsf1b, Sox11, App, Lepr, Serpine2, Egf, E2f1, Bcl2l1, Kif3a, Syn3, Dsp, Plcg1*), 6 in differentiation (*Dnmt1, Ptprf, Apoe, Serpine2, Egf, Rest*), 6 in survival (*Slc6a4, Dnmt1, Ptprf, App, Serpine2, Vipr2*), 2 in dendritogenesis (*Disc1, Apoe*), 1 in migration (*Disc1*), and 3 in maturation (*Disc1, Frzb, Apoe*) (Fig. 1A left, 1D upper panel). Furthermore, 11 genes are not associated with a specific event throughout adult neurogenesis (*Cacna1c, Dcc, Tpo, Esr2, Nefh, Mmp2, Mmp9, Jag1, Casp6, Cnga1, Fut4*) (Fig. 1D upper panel, *expression*). However, they are expressed in specific cell types during the neurogenic process (see below).

Regarding the cell stages defined by MANGO, 24 of the 33 genes with Cn SNPs are expressed in neural precursors (*Nf1, Cacna1c, Slc6a4, Lrp2, Dnmt1, Ptprf, Frzb, Apoe, Tnfrsf1b, Sox11, App, Lepr, Serpine2, Egf, Rest, E2f1, Bcl2l1, Kif3a, Syn3, Dsp, Plcg1, Casp6, Cnga1, Fut4*), with 7 of them specifically expressed in neural stem cells (Type 1) (*Dnmt1, Frzb, Apoe, App, Rest, Kif3a, Fut4*), 4 in undetermined progenitors (Type 2a) (*Apoe, Rest, Kif3a, Vipr2*), 1 in determined progenitors (Type 2b) (*Sox11*), and 1 in neuroblast-like cells (Type 3) (*Rest*) (Fig. 1A right, 1D inferior panel). Besides, 17 of the 33 genes are expressed in newborn differentiated neurons (*Disc1, Dcc, Slc6a4, Tpo, Dnmt1, Ptprf, Frzb, Apoe, Tnfrsf1b, Sox11, App, Lepr, Serpine2, Egf, E2f1, Vipr2, Nefh*). Among these genes, 8 are specifically expressed in immature neurons (*Disc1, Syn3, Mmp2, Mmp9*) or mature neurons (*Cacna1c, Frzb, Rest, Vipr2*). We identified 2 genes expressed in DCX-positive cells without any other specific cell stage markers, according to MANGO (*Esr2, Jag1*) (Fig. 1A right, 1D inferior panel).

Based on the gene expression profile of the mouse DG available in the Hipposeq platform^32^, 26 of the 33 genes with Cn SNPs were also found to be differentially expressed in mature granule cells (GC) (Supplementary Fig. S1C).

In a recent study, Artegiani et al. (2017) reported a gene expression profile that characterizes neural stem cells (NSC) (591 genes) and neural progenitors (1065 genes) in the mouse DG^33^. Based on that report, we investigated whether C58/J mice exhibited any Cn SNP in the signature genes of NSC and neural progenitors. We identified a total of 142 genes with at least one Cn SNP in C58/J mice, with 64 genes corresponding to the NSC genetic profile and 78 genes corresponding to the neural progenitors’ genetic profile (Fig. 1B, Supplementary Table S2). The *Fmn2* and *Zfp106* genes displayed the highest enrichment of Cn SNPs in the C58/J strain (Fig. 1E). Notably, three Cn SNPs in the *Fmn2* gene (rs31943453, rs31944226, rs215748054) and nine Cn SNPs in the *Zp106* gene (rs33851912, rs33203351, rs248533495, rs232926677, rs238741875, rs49978163, rs27422297, rs27422295, rs27422293) were found to be highly prone to damaging the protein structure based on impact predictions conducted using the PolyPhen-2 platform^31^ (Supplementary Table S3).

To obtain a more comprehensive understanding of the potential mechanisms affected by the genes with Cn SNPs identified in the C58/J strain, we performed a Gene Ontology (GO) enrichment analysis using the g:Profiler platform^34^.

**Figure 2:**
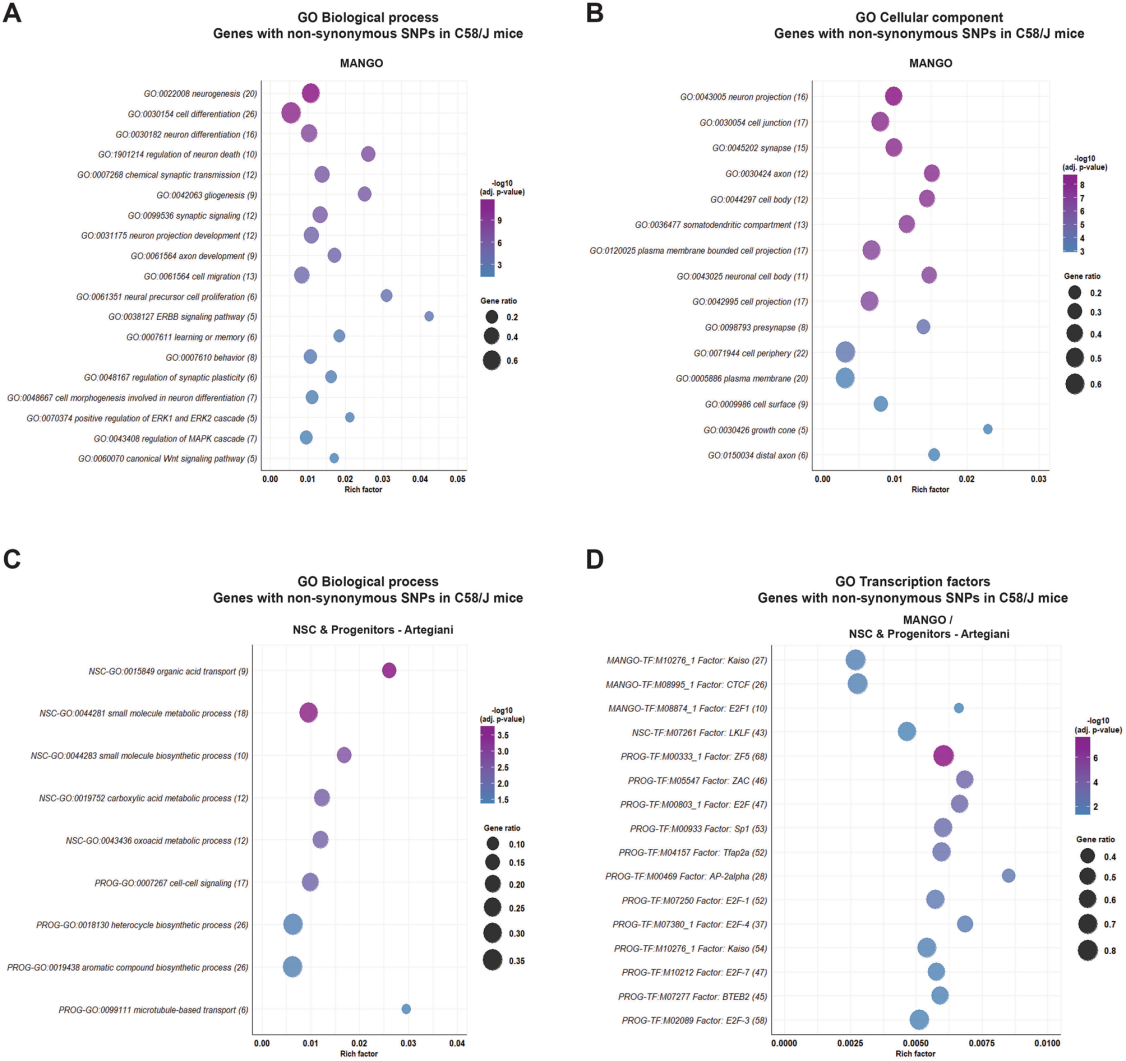
**Gene Ontology (GO) enrichment analysis for neurogenesis-associated genes with coding non-synonymous SNPs in the C58/J strain**. Enriched GO terms resulting from the enrichment analysis of the 33 genes with Cn SNPs associated with adult neurogenesis in C58/J mice, according to the MANGO database, in the *biological process* (**A**) and *cellular component* (**B**) categories. (**C**) Enriched GO terms resulting from the analysis of the 64 and 78 genes with Cn SNPs associated with NSC and neural progenitors (PROG) in C58/J mice, according to the Artegiani database, in the *biological process* category. (**D**) The genes with Cn SNPs in C58/J mice from both databases (MANGO and Artegiani) were enriched in DNA-binding motifs for specific transcription factors (TFs). Adjusted *p*-values were obtained through g:SCS multiple testing correction method on g:Profiler. The number of genes found in each GO term is indicated in parentheses. *Gene ratio:* the number of requested genes found in the functional category divided by the number of genes from the background genome. *Rich factor*: the number of requested genes found in the functional category divided by the number of total genes comprised in the specific functional category.

Among the top 20 enriched terms in the *biological process* category resulting from the GO enrichment analysis of the 33 genes with Cn SNPs in C58/J mice reported by MANGO, terms associated with the regulation of cell proliferation, development, nervous system development, neurogenesis, cell differentiation, cell signaling, and cell death were identified (Supplementary Fig. S1A, Supplementary Table S4). Other GO terms in the *biological process* category involved in the regulation of neurogenesis were enriched, including terms associated with neuron differentiation, neuron death, migration, neuron projection development, regulation of the ERBB, MAPK, and WNT signaling pathways, learning, memory, and behavior (Fig. 2A). Additionally, the enriched GO terms in the *cellular component* category were related to neuronal structures (Fig. 2B).

The GO enrichment analysis of the genes with Cn SNPs in C58/J mice corresponding to NSC and neural progenitors’ datasets showed enriched terms associated with organic acid metabolism and biosynthesis in NSC, and cell signaling and heterocycle compound biosynthesis in neural progenitors in the *biological process* category (Fig. 2C). GO terms associated with cell compartments and structural support, such the membrane, nucleoplasm, and cytoskeleton, were also enriched (Supplementary Fig. S1B, Supplementary Table S4).

The analysis on g:Profiler also revealed that the majority of genes with Cn SNPs in C58/J mice from each dataset (MANGO, NSC, and progenitors) possess DNA-binding motifs for specific transcription factors (*MANGO genes*: Kaiso, CTCF, and E2F1. *NSC genes*: LKLF. *Neural progenitors´ genes*: ZF5, ZAC, E2F, Sp1, Tfap2a, AP-2alpha, E2F-1/3/4/7, and BTEB2) reported by the TRANSFACT database ^34–36^ (Fig. 2D, Supplementary Table S4).

### The spatial distribution and morphology of newborn cells are atypical features in the DG of the C58/J strain

We investigated whether the C58/J model of autism exhibited alterations in the neurogenic process in the hippocampus based on the predicted changes in adult neurogenesis revealed by the in-silico analysis.

**Figure 3:**
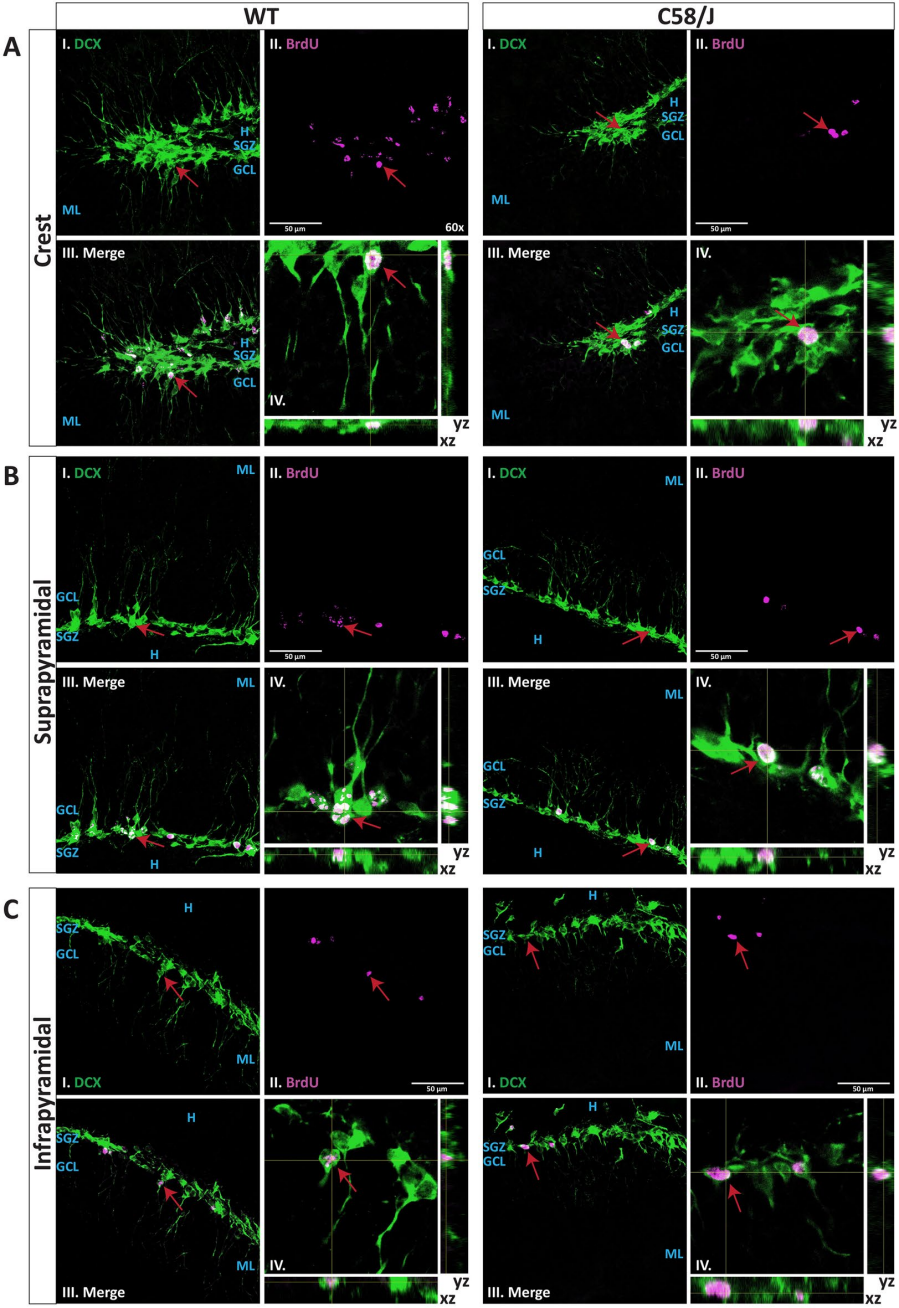
**Evaluation of BrdU + /DCX + newborn neurons in the DG of the C58/J and WT strains**. The representative confocal images show immunofluorescence for BrdU (magenta) and DCX (green) in cells of the DG from WT mice (left panel) and C58/J mice (right panel). The panels correspond to the crest (**A**), suprapyramidal (**B**), and infrapyramidal (**C**) blades in both strains. Each panel shows I) DCX channel, II) BrdU channel, III) merged channels, and IV) orthogonal xz and yz views of the BrdU + /DCX + cells indicated by red arrows. The DG layers (SGZ, GCL, ML, and hilus) are indicated in blue legends. The images are maximal intensity projections from 60x magnification z-stacks. The orthogonal views are shown as enlarged insets from the 60x magnification. Scale bars: 50 µm.

**Figure 4:**
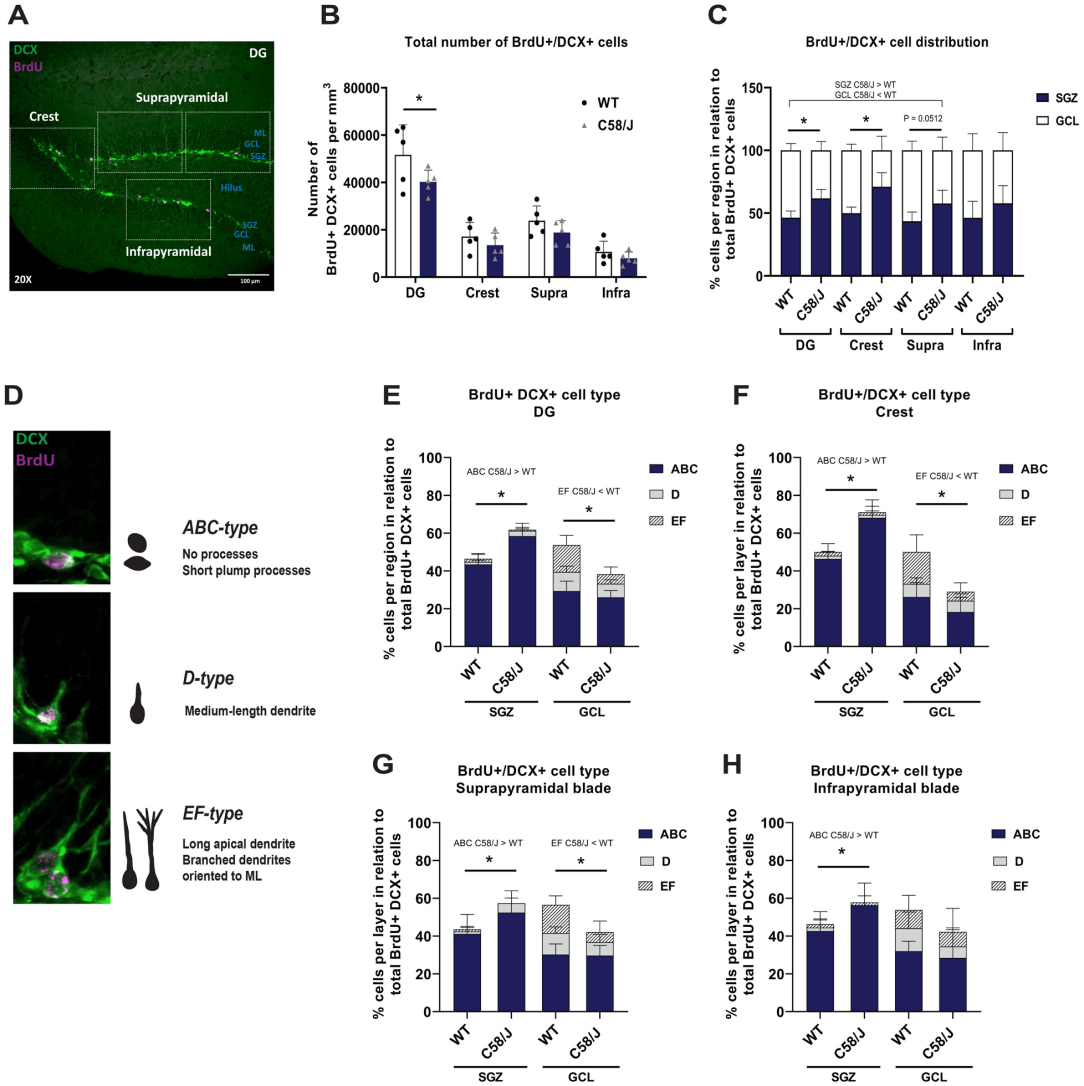
**Analysis of the number and morphology of BrdU + /DCX + cells in the C58/J and WT strains according to their distribution within DG regions and layers**. (**A**) A representation of the analyzed regions of interest (rectangles) in the crest, suprapyramidal and infrapyramidal blades in the dorsal DG from C57BL/6 J WT mice is shown (20x magnification. Scale bar: 100 µm). (**B**) Estimated total number of BrdU + /DCX + cells per mm^3^ in the whole DG, the crest, suprapyramidal (supra), and infrapyramidal (infra) blades from both strains. Two-way ANOVA followed by Sidak’s correction: ***DG****:* F_(1,32)_ = 7.620, *p = 0.0374. (**C**) Percentage of BrdU + /DCX + cells distributed within the subgranular zone (SGZ) and the granule cell layer (GCL) of the whole DG, the crest, suprapyramidal (supra), and infrapyramidal (infra) blades from both strains. Two-way ANOVA followed by Sidak’s correction: ***DG-SGZ:*** F_(1, 16)_ = 8.609, *p = 0.0025; ***DG-GCL:*** *p = 0.0025; ***Crest-SGZ:*** F_(1, 16)_ = 29.43, *p = 0.0028; ***Crest-GCL:*** *p = 0.0028; ***Supra-SGZ:*** F_(1,16)_ = 0.08987, *p* = 0.0512; ***Supra-GCL:**** p* = 0.0512. (**D**) Representative examples of BrdU + /DCX + cells categorized as ABC-type, D-type, and EF-type. Percentage of BrdU + /DCX + cells classified according to their morphology within the subgranular zone (SGZ) and the granule cell layer (GCL) of the whole DG (**E**), the crest (**F**), suprapyramidal (**G**), and infrapyramidal (**H**) blades from both strains. Two-way ANOVA followed by Sidak’s correction: (**E**) ***SGZ-ABC*:** F_(1, 24)_ = 13.35, *p < 0.0001; ***GCL-EF*:** F_(1, 24)_ = 12.28**,** *p = 0.0039; (**F**) ***SGZ-ABC*:** F_(1, 24)_ = 15.32, *p < 0.0001; ***GCL-EF*:** F_(1, 24)_ = 9.102, *p = 0.0185; (**G**) ***SGZ-ABC*:** F_(1, 24)_ = 3.721, *p = 0.0351; ***GCL-EF*:** F_(1, 24)_ = 7.023, *p = 0.0171; (**H**) ***SGZ-ABC*:** F_(1, 24)_ = 2.385, *p = 0.0125. All results are expressed as mean ± SD. n = 5 animals per group.

We evaluated the number of newborn cells in the dorsal dentate gyrus (DG) of young adult C58/J and WT (C57BL/6J) mice. Using confocal microscopy, we detected newly generated neuronal-lineage cells (BrdU + /DCX +) that had undergone proliferation, survival, and differentiation within a 2-weeks period between the BrdU administration protocol and the time of sacrifice (Fig. 3). To obtain a representative sample of the number of double-positive cells in the dorsal DG of both groups, we imaged and analyzed one region of interest (ROI) from the crest, two from the suprapyramidal blade, and one from the infrapyramidal blade (Figs. 3 and 4A) (See *methods* section for detailed analysis procedures).

First, we estimated the total number of BrdU + /DCX + cells in the DG of WT and C58/J mice. We observed a lower number of double-positive cells in the whole DG of C58/J mice compared to the WT group (Fig. 4B, DG: *p = 0.0374, WT: 51,657.4 ± 12,723.6 vs. C58/J: 40,259.3 ± 4987.6 cells per mm^3^). However, when we analyzed DG regions separately, we found no significant differences in the number of double-positive cells in the crest, and the suprapyramidal and infrapyramidal blades comparing both strains (Fig. 4B, *crest, supra, infra*).

The spatial positioning of the young neurons within the layers of the DG has been associated with their migration ability and maturation stage. Hence, we examined the distribution of BrdU + /DCX + cells within the subgranular zone (SGZ), granular cell layer (GCL), molecular layer (ML), and hilus of the DG (Supplementary Fig. S2A–D). Since only one double-positive cell was found in the ML of C58/J mice, we excluded this layer from the analysis.

There was a diminished number of BrdU + /DCX + cells within the GCL of the whole DG in the C58/J strain compared to WT mice (Supplementary Fig. S2A, GCL: *p = 0.0003, WT: 26,665.2 ± 7801.9 vs. C58/J: 14,116.9 ± 2741.8 cells per mm^3^). We did not find any differences in the number of double-positive cells within the SGZ and the hilus between both groups (Supplementary Fig. S2A, *SGZ, hilus*). When we analyzed the DG by region, we observed that the number of double-positive cells was lower within the GCL of the suprapyramidal blade in the C58/J mice (Supplementary Fig. S2C, GCL: *p = 0.0339, WT: 12,966.6 ± 4,073.5 vs. C58/J: 7424.4 ± 2,435.6 cells per mm^3^). We did not observe any changes in the number of double-positive cells within the SGZ or the GCL from the crest or from the infrapyramidal blade (Supplementary Fig. S2B, D).

To assess whether changes in the distribution of BrdU + /DCX + cells within the SGZ and GCL could not solely be attributed to the lower number of double-positive cells in the DG of C58/J mice, we calculated the proportion of BrdU + /DCX + cells present in each layer in relation to the total number of double-positive cells in the whole DG or in each specific region (Fig. 4C).

In the whole DG, the percentage of BrdU + /DCX + cells confined to the SGZ was higher in the C58/J strain compared to WT mice (Fig. 4C, DG-SGZ: *p = 0.0025, WT: 46.4 ± 5.3 vs. C58/J: 61.8 ± 7.0% of cells). In contrast, the proportion of double-positive cells within the GCL was reduced in the model of autism (Fig. 4C, DG-GCL: *p = 0.0025, WT: 53.6 ± 5.3 vs. C58/J: 38.1 ± 7.0% of cells). Similarly, in the crest of C58/J mice, a majority of DCX + /BrdU + cells were found within the SGZ, while a lower percentage of them was within the GCL (Fig. 4C, Crest-SGZ: *p = 0.0028, WT: 49.9 ± 4.9 vs. C58/J: 71.0 ± 11.2; *Crest-GCL:* *p = 0.0028, WT: 50.0 ± 4.9 vs. C58/J: 28.9 ± 11.2% of cells). We observed a similar trend in the SGZ and the GCL of the suprapyramidal blade in the C58/J strain, although it did not reach statistical significance (Fig. 4C, *Supra-SGZ: p* = 0.0512, WT: 43.5 ± 7.3 vs. C58/J: 57.7 ± 10.6; *Supra-GCL:* *p* = 0.0512, WT: 56.5 ± 7.4 vs. C58/J: 42.3 ± 10.6% of cells). There were no changes in the ratio of BrdU + /DCX + cells within the SGZ and GCL of the infrapyramidal blade when comparing both strains (Fig. 4C, *Infra*).

Changes in the distribution of BrdU + /DCX + neurons within the SGZ and GCL of C58/J mice may be linked to the maturation stage of the cells. Therefore, we categorized BrdU + /DCX + cells based on their morphology, as cell shape modifications are closely associated with the maturity stage of new neuroblasts transitioning into granule cells^21^. We classified cells as early neuroblasts (*ABC-type*) if they exhibited short, plump dendritic processes or none. Cells in an intermediate maturation state corresponding to the *D-type* were those with a medium-length vertical dendrite extending towards the molecular layer (ML). Lastly, cells with the most mature morphology, characterized by a long apical dendrite or/and at least one branched dendrite oriented toward the ML, were classified as *EF-type* (Fig. 4D).

We estimated the total number of ABC, D, and EF-type BrdU + /DCX + cells within each layer (SGZ, GCL) and region (crest, suprapyramidal and infrapyramidal blades) of the DG (Supplementary Fig. S2E-L). In the whole DG of C58/J mice, there was a lower number of ABC and EF-type BrdU + /DCX + cells within the GCL (Supplementary Fig. S2F, *GCL-ABC*: *p = 0.0106, WT: 14,953.4 ± 5,589.8 vs. C58/J: 9,515.8 ± 437.4; GCL-EF: *p = 0.0193, WT: 6,901.6 ± 2,598.5 vs. C58/J: 1,882.2 ± 1,506.3 cells per mm^3^), but no differences within the SGZ compared to WT mice (Supplementary Fig. S2E). When we analyzed the DG by region, we observed a lower number of EF-type cells within the GCL of the suprapyramidal blade (Supplementary Fig. S2J, *GCL-EF*: *p = 0.0327, WT: 3,450.8 ± 1,718.2 vs. C58/J: 836.6 ± 792.9 cells per mm^3^), and a reduced number of ABC-type cells within the GCL of the infrapyramidal layer of C58/J mice (Supplementary Fig. S2L, *GCL-ABC*: *p = 0.0386, WT: 3,450.8 ± 1,977.2 vs. C58/J: 1,673.1 ± 935.3 cells per mm^3^). We did not observe changes in the number of any cell type within the SGZ and GCL of the crest, and the SGZ of the suprapyramidal and infrapyramidal blades comparing both strains (Supplementary Fig. S2G–I, K).

Then, to evaluate whether differences in the number of BrdU + /DCX + cells with a specific cell type could not be solely attributed to the lower number of double-positive cells in the DG of C58/J mice, we determined the proportion of ABC, D, and EF-type cells in relation to the total number of double-positive cells found within the SGZ and the GCL in the whole DG and in each region (Fig. 4E–H).

In the whole DG of the C58/J strain, there was a higher proportion of ABC-type cells within the SGZ and a reduced ratio of EF-type cells within the GCL compared to the WT group (Fig. 4E, *SGZ-ABC*: *p < 0.0001, WT: 43.4 ± 5.8 vs. C58/J: 58.4 ± 6.8; *GCL-EF*: *p = 0.0039, WT: 14.2 ± 5.2 vs. C58/J: 4.9 ± 3.9% of cells). Similarly, when we analyzed the crest and each DG blade separately, we observed a higher proportion of ABC-type cells within the SGZ and a lower proportion of EF-type cells within the GCL of the crest (Fig. 4F, *SGZ-ABC*: *p < 0.0001, WT: 46.3 ± 3.9 vs. C58/J: 68.1 ± 9.5; *GCL****-EF*:** *p = 0.0185, WT: 16.8 ± 9.0 vs. C58/J: 4.8 ± 4.7% of cells) and the suprapyramidal blade (Fig. 4G, *SGZ-ABC*: *p = 0.0351 WT: 41.1 ± 10.3 vs. C58/J: 52.34 ± 11.6; *GCL-EF*: *p = 0.0171, WT: 14.9 ± 4.8 vs. C58/J: 5.4 ± 5.8% of cells) of C58/J mice. Additionally, there was a higher proportion of ABC-type cells within the SGZ of the infrapyramidal blade in the C58/J strain (Fig. 4H, *SGZ-ABC*: *p = 0.0125, WT: 42.6 ± 10.3 vs. C58/J 56.2 ± 11.7% of cells), but we did not observe any changes in other cell types or within the GCL when comparing both groups. The proportion of D-type cells was not different within the SGZ or the GCL in the whole DG, the crest, or DG blades when comparing both strains (Fig. 4E–H, *SGZ-D, GCL-D*).

### Genes associated with neurogenesis carrying non-synonymous SNPs in the C58/J strain are orthologous to human genes implicated in ASD

Finally, to gain insights into the relevance of the changes in adult DG neurogenesis found in the C58/J strain, we focused on evaluating the implication of all the identified genes with Cn SNPs (Supplementary Table S2) in the etiology of ASD. Thus, the genes with Cn SNPs in the C58/J mice were searched for human orthologous genes in the SFARI GENE database^37^, which provides current information on genes associated with ASD**.**

Among the genes with Cn SNPs in C58/J mice reported by MANGO, we identified 8 genes with reported human orthologs in the SFARI database (Supplementary Table S5). *NF1* and *CACNA1C* exhibited the highest association scores (score 1) for ASD (Table 1). Moreover, we identified 15 genes with Cn SNPs from the NSC and progenitors’ dataset with human orthologs in SFARI (Supplementary Table S5). Among these, *ANK2, RFX3, CHD3, MYT1L, PHIP,* and *PHF21A* display the highest association scores (score 1) for ASD (Table 1). *MYT1L* and *RFX3* were also ranked as strong candidates specifically for ASD, rather than other neurodevelopmental disorders, according to the EAGLE score^37,38^. *NF1* and *ANK2* were scored by EAGLE as moderate candidates (Table 1).

**Table 1:** Genes with Cn SNPs involved in adult neurogenesis in C58/J mice are orthologous to human genes associated with autism spectrum disorder (high score according to SFARI).

Gene symbol	Gene name	Human orthologous gene symbol	SFARI gene score	EAGLE score	Database
*Nf1*	Neurofibromatosis 1	*NF1*	1	9.3	MANGO
*Cacna1c*	Calcium channel, voltage-dependent, L type, alpha 1C subunit	*CACNAC1C*	1	NA	MANGO
*Chd3*	Chromodomain helicase DNA binding protein 3	*CHD3*	1	NA	NSC & neural progenitors (Artegiani)
*Myt1l*	Myelin transcription factor 1-like	*MYT1L*	1	20.35	NSC & neural progenitors (Artegiani)
*Phf21a*	PHD finger protein 21A	*PHF21A*	1	5.35	NSC & neural progenitors (Artegiani)
*Ank2*	Ankyrin 2	*ANK2*	1	10.8	NSC & neural progenitors (Artegiani)
*Rfx3*	Regulatory factor X, 3	*RFX3*	1	15.95	NSC & neural progenitors (Artegiani)
*Phip*	Pleckstrin homology domain interacting protein	*PHIP*	1	NA	NSC & neural progenitors (Artegiani)

*NA *Not available data.

Recently, Kim and collaborators reported a group of genes that were differentially expressed in radial glial cells (Pax6 + or Vimentin +) from the prefrontal cortex of individuals with ASD^39^. Based on this information, we identified 31 genes with Cn SNPs in C58/J mice associated with adult neurogenesis that were orthologous to the genes dysregulated in the radial glial cells of individuals with autism (Supplementary Table S2-2.4).

## Discussion

Dysregulation in the neurogenic process is one of the underlying mechanisms associated with the neurobiological changes found in ASD^4,39^. Individuals with autism often exhibit atypical hippocampal anatomy and connectivity throughout their lifespan, which may be associated with alterations in the hippocampal neurogenic process^40^.

The adult neurogenic process in the mouse hippocampus is controlled by gene expression patterns and is strongly influenced by polymorphisms^17,22,23^. Therefore, we used an in-silico approach to identify single-nucleotide polymorphisms (SNPs) in genes relevant to adult hippocampal neurogenesis in the C58/J model of idiopathic autism.

Our results show that C58/J mice display non-synonymous SNPs (Cn SNPs) in 33 genes involved in the adult neurogenic process, according to the MANGO database. The majority of these genes are implicated in the regulation of proliferation, differentiation, and survival of neural precursors. This suggests that the C58/J strain may exhibit impairments in the early events of the hippocampal neurogenic process, as previously proposed for cortical neurogenesis in ASD^8,9,41,42^.

In line with this, C58/J mice also present Cn SNPs in 64 and 78 genes, corresponding to the signature genetic profile of NSC and neural progenitors, respectively, as reported by Artegiani et al.^33^. These Cn SNPs may interfere with the genetic regulation of NSCs during their transition from a dormant to a proliferative stage and subsequent differentiation into neural progenitors. Evidence of this is observed in *Shank3b* knockout mice, a model of genetic autism, where altered gene expression in NSCs resulted in increased quiescence and reduced neuroblast differentiation frequency^39^.

Our GO enrichment analysis of genes with Cn SNPs revealed enriched terms related to cell proliferation and the regulation of MAPK and WNT signaling pathways. Interestingly, experiments with iPSCs-derived neural progenitor cells from individuals with ASD have shown that dysregulation of cell proliferation is associated with alterations in the RAS/ERK and WNT signaling pathways^4,6^.

Other enriched GO terms were associated with metabolic processes and neuron death, critical mechanisms for neurogenesis progression and homeostasis^16,33,43^. We also identified genes with Cn SNPs that are important for later events in neurogenesis, such as migration, dendritogenesis, and maturation, according to the MANGO database. Additionally, we found enriched GO terms potentially involved in neuronal integration, including synaptic transmission and plasticity, as well as terms associated with neural processes that can be modulated by hippocampal adult neurogenesis, such as learning, memory, and behavior^12,44,45^. The genes with Cn SNPs were also found to be expressed in mature granule cells (GC) according to Hipposeq platform, suggesting their potential relevance for GC functionality.

Hence, these in-silico predictions based on the genetic variants of C58/J mice may imply that adult neurogenesis in the hippocampus is dysregulated at different stages of the neurogenic process: during the proliferation of NSC and neural progenitors, neuronal differentiation, neuronal death, migration, and morphological and functional maturation of neuroblasts into GC.

The GO enrichment analysis based on the TRANSFACT database revealed that the genes with Cn SNPs in C58/J mice possess binding motifs for specific transcription factors (TFs). This suggests that many of these genes may share regulatory pathways to control their expression during the adult neurogenic process. However, there is limited evidence regarding the specific participation of these TFs, such as E2F1 and Sp1, in the regulation of gene expression during adult neurogenesis^46,47^.

Interestingly, one of the genes with a high number of Cn SNPs in C58/J mice, *Disc1* (Disrupted-in-schizophrenia 1), has a strong participation in proliferation, morphogenesis, differentiation, and migration during hippocampal adult neurogenesis^48^. Another gene, *Fmn2* (Formin-2), also showed enrichment with Cn SNPs and is essential for promoting neural progenitor proliferation^49^. Moreover, given that the *Disc1* and *Fmn2* genes carry Cn SNPs predicted to significantly damage the protein structure, it is possible that the function of both proteins is compromised in the C58/J strain. Therefore, future studies should include functional analyses to assess the potential protein alterations.

We further tested whether the in-silico predicted changes in adult neurogenesis of the C58/J strain could be related to in vivo hippocampal neurogenesis by analyzing the number and distribution of BrdU + /DCX + cells in young adult (6-weeks-old) C58/J mice compared to the WT strain C57BL/6J.

In agreement with the Cn SNPs we identified in genes involved in cell proliferation, differentiation, and survival, we observed an overall lower number of BrdU + /DCX + cells in the DG of C58/J mice. This suggests a reduced rate of neuronal-lineage cells (Type 2b and Type 3 cells) that underwent proliferation and differentiation, or that had a lower survival rate within the 2-weeks evaluation period. However, since DCX only allows us to identify Type 2b progenitors and Type 3 neuroblasts, we cannot rule out the possibility of impaired proliferation in NSC (Type 1 cells) and neural intermediate progenitors (Type 2a cells). Additionally, cell death also plays a crucial role in regulating the number of neuroblasts that survive and mature. Therefore, further experiments in autism models are required to evaluate the populations of NSC and Type 2a cells, as well as the death rate of hippocampal newborn neurons.

Maturation trajectories of newborn neurons are associated with their migration from the SGZ to the GCL, accompanied by morphological modifications^16,21^. The morphological classification of newborn cells in C58/J mice indicated a high prevalence of immature *ABC-type* cells within the SGZ, which may correspond to Type 2b progenitors and early neuroblasts that remained close to their site of origin. Consistent with this, the C58/J strain exhibited a reduced proportion of *EF-type* cells in the GCL, which may represent late neuroblasts with morphological maturation signs, such as the outgrowth of apical branched dendrites and migration to the GCL. These observed changes may be associated with a delay in the maturation trajectory of newborn neurons in the DG of C58/J mice, potentially linked to the predicted alterations in the expression of relevant genes involved in hippocampal neurogenesis.

Nevertheless, it is important to point out that DCX-expressing newborn neurons with complex morphological features do not represent cells in a fully mature state. Therefore, in future experiments, we will evaluate further maturation steps by sacrificing the animals at later time points after BrdU administration and by analyzing the expression of proteins that appear after DCX such as calretinin and calbindin. Additionally, we will further characterize the electrophysiological properties of these new neurons to analyze their potential incorporation into the established circuits of mature granule cells in the DG.

The C58/J strain may not exhibit changes in the migration trajectory of newborn neurons, as we did not observe differences in the number of BrdU + /DCX + cells within the hilus, a common characteristic of ectopic migration^50^. However, we cannot rule out the possibility of an altered migration ability of newborn cells in C58/J mice, considering the presence of Cn SNPs in the *Disc1* gene. Proper migration of newly born neurons in the mouse DG relies on *Disc1* expression during both hippocampal development and adult neurogenesis^51–53^.

Furthermore, we observed differential changes in the distribution and morphological maturation of neuroblasts in C58/J mice depending on the region of the DG (the crest, the suprapyramidal and infrapyramidal blades). It has been proposed that topographic variations in neurogenesis along the transverse axis of the DG (suprapyramidal vs. infrapyramidal blades) are necessary for the formation of a gradient of immature neurons/mature granule cells, which is involved in the specific organization of hippocampal neural circuits^54–56^.

Alterations in hippocampal neurogenesis have been found in other murine models of autism^40,57^. For instance, the BTBR strain exhibits a reduced number of hippocampal newborn neurons^58^, and the valproic acid (VPA)-induced mouse model shows a decreased ratio of neuronal differentiation in the hippocampus^59^. Interestingly, in the *Auts2* knockout mice, the induction of the expansion of hippocampal newborn neurons rescued impaired social novelty recognition^60^.

Finally, we investigated whether changes in hippocampal adult neurogenesis in the C58/J model could be linked to ASD etiology by searching for orthologous human genes previously associated with autism. According to the SFARI GENE database^37^, we identified 22 ASD-related genes that are orthologous to the genes with variants in C58/J mice involved in adult neurogenesis. Among them, 8 genes showed a high score for ASD association. Additionally, we identified 31 genes with Cn SNPs in C58/J mice that were orthologous to genes dysregulated in the radial glial cells of individuals with autism, as reported by Kim et al.^39^.

In conclusion, these results suggest that the C58/J model of idiopathic autism exhibits a set of genes with non-synonymous SNPs associated with the regulation of adult neurogenesis, some of which have human orthologous genes previously implicated in ASD. These genetic variants could contribute to the observed alterations in adult neurogenesis in the DG of C58/J mice. Thus, the dysregulation of the neurogenic process in the juvenile hippocampus should be further evaluated as a contributing mechanism involved in the etiology of ASD.

## Methods

### Animals

Two female and three male mice (n = 5 animals per group), 4 weeks old, from C57 BL/6J and C58/J strains, were used for the in vivo experiments. The animals were purchased from The Jackson Laboratory (BHB, ME, USA). The litters, grouped by sex, were housed in individual cages and kept on a commercial pelletized diet (T.G. rodent diet T2018S.15, Envigo), ad libitum. They were maintained under a reversed 12:12 h light/dark cycle, with lights on from 19:00 to 7:00.

### Ethics approval and consent to participate

All animal procedures were carried out following the ARRIVE Guidelines ^61^ and were performed in agreement with local government rules (Official Mexican Standard NOM-062-ZOO-1999) and the local Institutional Animal Care and Research Advisory Committee (CICUAL, ID 189 and 10367) from the Universidad Nacional Autónoma de México (UNAM)**.**

### BrdU administration

Four-week-old mice received an i.p. injection of BrdU (5-bromo-2-deoxyuridine; Sigma) (50 mg/kg) for three consecutive days. BrdU was dissolved in 0.9% NaCl solution and was preheated at 60 °C immediately before administration. Animals were sacrificed and perfused 2 weeks after the administration of the first dose.

### Histological procedures

Animals were anesthetized with sodium pentobarbital (210 mg/kg) and intracardially perfused with chilled 0.9% NaCl solution followed by chilled 4% paraformaldehyde (PFA) prepared in phosphate buffer (0.1 M, pH 7.4). Brains were removed and immersed into 15% and 30% sucrose dissolved in 0.1 M phosphate buffer.

Brains were sliced into 40 μm-coronal sections using a cryostat (MEV, SLEE Medical GMBH, Mainz, Germany) and stored at 4 °C while immersed in a cryoprotectant solution (25% ethylene glycol, 25% glycerol, 50% 0.2 M PB, pH 7.4) until processed for immunofluorescence.

### Immunofluorescence protocol

Four serial sections (spaced by 160 μm) from the dorsal dentate gyrus (DG) (AP coordinates respective to Bregma: − 1.7 to − 2.27 mm)^62^ of each experimental animal were selected and washed with PBS (0.1 M, pH 7.4). All following steps were performed under agitation. The selected free-floating sections were permeabilized in PBS–Triton X 0.3% for 30 min. Antigen retrieval was performed using citrate buffer solution (10 mM, pH 6) at 60–70 °C for 20 min, followed by an additional 20 min using fresh solution. For BrdU detection, sections were denatured in 2N HCl at 37 °C for 30 min, then neutralized with borate buffer (0.1 M, pH 8.5). After washing with PBS, blocking was performed with 1% horse serum in PBS-Triton X 0.3% for 1 h. Subsequently, the sections were incubated with primary antibodies: mouse anti-BrdU (#Cat: 11,170,376,001, Roche, dilution 1:100) and goat anti-DCX (#Cat, Santa Cruz Biotechnology, dilution 1:200) for 48 h at 4 °C. The sections were washed with PBS and then incubated with secondary antibodies: Alexa Fluor 488-conjugated donkey anti-mouse (#Cat: A-21202, Invitrogen, dilution 1:300) and DyLight 594-conjugated donkey anti-goat (#Cat: 53,044, Jackson ImmunoResearch, dilution 1:300) for 2 h at room temperature. The sections were mounted on slides and protected with DAKO fluorescent mounting medium (DAKO, Carpinteria, CA, USA) before placing a coverslip.

### Image acquisition.

A total of 5 brains from the C57BL/6J strain and 5 brains from the C58/J strain were analyzed. The dorsal DG from each of the selected 4-section series per animal was imaged using confocal microscopy (Nikon A1R + STORM confocal microscope). We acquired 19–22 z-stack images (1.2 µm separation step between optical sections) at 60x magnification (WI objective, 2048 × 2048 pixels resolution). To recollect a representative sample of the whole DG from both groups, we imaged one field from the crest, two from the suprapyramidal blade, and one from the infrapyramidal blade.

### Analysis of the number of BrdU + /DCX + cells and classification by morphological type

The confocal acquired images were exported to Fiji 2.9.0 software^63^ to merge 594 nm (DCX) and 488 nm (BrdU) color channels in order to detect the BrdU + /DCX + cells.

The analysis of double-positive cells was performed on individual 60 × optical sections from z-stacks ranging from 9.6 to 13.2 µm each. Care was taken to have a similar average number of optical slices per group. We analyzed one region of interest (ROI) from the crest, two from suprapyramidal blade, and one from the infrapyramidal blade. Cells were considered double-positive if they displayed colocalization of BrdU and DCX in at least three consecutive focal planes within the analyzed z-stack.

The number of BrdU + /DCX + cells was quantified in the crest, suprapyramidal, and infrapyramidal blades of the DG. We also evaluated the distribution of BrdU + /DCX + cells within the layers of the DG: the subgranular zone (SGZ), the granular cell layer (GCL), the molecular layer (ML), and hilus.

#### Semi-stereological estimation of total cell numbers

Then, we performed a semi-stereological estimation of the total number of double-positive cells per mm^3^ of the whole DG and its regions. The estimation was based on the optical fractionator equation as reported by Keuker et al.^64^:

$$N= \sum {Q}^{-}\cdot \frac{1}{ssf}\cdot \frac{1}{asf}\cdot \frac{1}{tsf}$$

where *N* is the estimated total number of cells, *Q-* is the total cell count, *ssf* is the section sampling fraction, *asf* is the area sampling fraction, and *tsf* is the thickness sampling fraction.

We calculated the mean group coefficient of error (CE) of our sampling scheme (Supplementary Table S6), which should be under 0.10 as indicated by Keuker et al.^64^.

The analyzed volume (mm^3^) of the DG was estimated based on the Cavalieri’s principle^65^:

$$V=T \cdot \sum {A}_{i}$$

where *V* is the estimated volume, *T* is the inverse of section sampling fraction multiplied by the section thickness, and *Ai* is the analyzed area of each section.

#### Morphological classification

To evaluate the morphology of BrdU + /DCX + cells, we applied the criteria established by Plümpe et al.^21^ with some modifications. We classified cells as ABC-type if they displayed short, plump dendritic processes or none. D-type cells were characterized by the presence of a medium-length vertical dendrite extending towards the molecular layer (ML). EF-type cells were identified by the presence of a long apical dendrite or/and at least one branched dendrite oriented towards the ML.

Both the quantification and classification analyses were performed under double-blind conditions.

### In silico evaluation of single-nucleotide polymorphisms (SNPs) in neurogenesis-associated genes.

We consulted the information on the 397 genes associated with adult neurogenesis available in the Mammalian Adult Neurogenesis Gene Ontology (MANGO) database^17^. MANGO is an open-access platform that systematically integrates curated data on neurogenesis obtained from published reports in mammals, including adult rodents from both sexes, using Gene Ontology (GO) principles. It provides a genetic profile associated with the various events and cell stages that comprise the adult neurogenic process^17^ (See Supplementary Table S1 for MANGO criteria and definitions).

In addition, we consulted the gene dataset previously reported by Artegiani et al.^33^. This dataset provides a genetic characterization of neural stem cells (NSC) and neural progenitors from the neurogenic niche in the DG of 6–10-weeks-old female and male mice, obtained through single-cell RNA sequencing (data of the genes differentially expressed in NSC and neural progenitors is available in the supplementary material from the work of Artegiani)^33^.

To investigate whether genes associated with adult neurogenesis carry single-nucleotide polymorphisms (SNPs) in the genome of the C58/J model of autism, the MANGO, NSC, and progenitor’s gene datasets were submitted to the *SNP data retrieval utility tool* of the Mouse Phenome Database (MPD)^28,66^. We used the Sanger4 Dataset (*Sanger SNP and indel data, 89* + *million locations, 37 inbred strains of mice, 2017*)^29^ to detect coding non-synonymous (Cn) SNPs, leading to changes in the coding amino acid sequence, in both adult females and males of the C58/J strain in comparison with the control C57 BL/6J strain.

Subsequently, we performed a Gene Ontology (GO) enrichment analysis using the g:Profiler platform (version e108_eg55_p17_9f356ae)^34^ to identify additional biological functions associated with the neurogenesis-related genes with Cn SNPs in C58/J mice. The GO enrichment analysis covered the *Biological process* and *Cellular componen*t categories. We also used the TRANSFACT database to detect DNA-binding motifs for transcription factors^34–36^.

The potential impact on the protein structure of Cn SNPs was predicted using the PolyPhen-2 platform^31^. PolyPhen-2, trained by the Humdiv dataset model, extracts various sequence and structure-based features of an amino acid substitution to perform a probabilistic classification of the functional significance of an allele replacement. This classification is based on pairs of false positive rate (FPR) thresholds: *benign* (indicating a low probability of protein damage), *possibly damaging* (indicating a less confident prediction of protein damage), or *probably damaging* (a more confident prediction of protein damage)^31^.

To evaluate the expression of genes with Cn SNPs in the granule cells of the mouse DG, we used the Hipposeq platform containing the gene expression characterization of excitatory cell classes in the hippocampal formation of mice from both sexes^32^. An analysis for multiple genes expression based on the *Dorsal–ventral survey of hippocampal principal cells dataset*^32^ was requested. The selected cell population was *Dorsal DG granule cells*. Data was retrieved as FPKM (fragments per kilobase of transcript per million reads mapped).

To identify whether genes with Cn SNPs display human orthologous genes implicated in the etiology of ASD, we searched for genes associated with autism spectrum disorder in the SFARI GENE database^37^ containing relevant up-to-date information. In addition, we searched for these genes in the repository reported by Kim and collaborators, which contains a group of genes that were found to be differentially expressed in radial glial cells (Pax6 + or Vimentin +) from the prefrontal cortex of individuals with ASD in comparison to neurotypical subjects^39^.

### Statistical analysis

The data obtained from the quantification of BrdU + /DCX + cells from both strains passed the Shapiro–Wilk test for normal distribution.

To compare the estimated number and percentage of BrdU + /DCX + cells based on their distribution within DG regions (crest, suprapyramidal and infrapyramidal blades), layers (SGZ, GCL, and hilus), and their morphological classification (ABC, D, and EF types) between control and C58/J mice, a two-way Analysis of Variance (ANOVA) was performed, followed by Sidak’s correction for multiple comparisons. A* p*-value below 0.05 was considered statistically significant. The results were plotted and statistically analyzed using RStudio 4.2.3 and GraphPad Prism 8.0.2 software.

Data of the quantification of BrdU + /DCX + cells were also analyzed by comparing both sexes within each strain, but we did not observe statistically significant changes depending on the sex effect (Two-way ANOVA followed by Sidak’s correction).

For the GO enrichment analysis, g:Profiler (version e108_eg55_p17_9f356ae) considers a result statistically significant if it corresponds to an experiment-wide threshold of a = 0.05 after applying the g:SCS multiple testing correction method, as reported by the platform^34^. The enriched GO terms were plotted using the log10 of *p*-adjusted values. The *Gene Ratio* was calculated by dividing the number of requested genes found in the functional category by the number of genes in the background genome (experimental gene set). The *Rich Factor* was calculated by dividing the number of requested genes found in the functional category by the total number of genes within that specific functional category.

### Supplementary Information

Supplementary Information.

Supplementary Table S1.

Supplementary Table S2.

Supplementary Table S3.

Supplementary Table S4.

Supplementary Table S5.

Supplementary Table S6.

## Data Availability

All data generated or analyzed during this study are included in this published article (and its Supplementary Information files).

## References

[1] American Psychiatric Association (2022). Diagnostic And Statistical Manual Of Mental Disorders, Fifth Edition, Text Revision (DSM-5-TR).

[2] World Health Organization. Autism. *Fact sheets: Autism* https://www.who.int/news-room/fact-sheets/detail/autism-spectrum-disorders (2023).

[3] Willsey HR, Willsey AJ, Wang B, State MW (2022). Genomics, convergent neuroscience and progress in understanding autism spectrum disorder. Nat. Rev. Neurosci..

[4] Courchesne E (2019). The ASD Living Biology: From cell proliferation to clinical phenotype. Mol. Psychiatry.

[5] Stoner R (2014). Patches of disorganization in the neocortex of children with autism. N. Engl. J. Med..

[6] Marchetto MC (2017). Altered proliferation and networks in neural cells derived from idiopathic autistic individuals. Mol. Psychiatry.

[7] Connacher R (2022). Autism NPCs from both idiopathic and CNV 16p11. 2 deletion patients exhibit dysregulation of proliferation and mitogenic responses. Stem Cell Rep..

[8] Adhya D (2021). Atypical neurogenesis in induced pluripotent stem cells from autistic individuals. Biol. Psychiatry.

[9] Cederquist GY (2020). A multiplex human pluripotent stem cell platform defines molecular and functional subclasses of autism-related genes. Cell Stem Cell.

[10] Gage FH (2019). Adult neurogenesis in mammals. Science (80-).

[11] Kempermann G, Song H, Gage FH (2015). Neurogenesis in the adult hippocampus. Cold Spring Harb. Perspect. Biol..

[12] Snyder JS (2019). Recalibrating the relevance of adult neurogenesis. Trends Neurosci..

[13] Li Y, Shen M, Stockton ME, Zhao X (2019). Hippocampal deficits in neurodevelopmental disorders. Neurobiol. Learn. Mem..

[14] Saitoh O, Karns CM, Courchesne E (2001). Development of the hippocampal formation from 2 to 42 years: MRI evidence of smaller area dentata in autism. Brain.

[15] Wegiel J (2010). The neuropathology of autism: Defects of neurogenesis and neuronal migration, and dysplastic changes. Acta Neuropathol..

[16] Abbott LC, Nigussie F (2020). Adult neurogenesis in the mammalian dentate gyrus. Anat. Histol. Embryol..

[17] Overall RW, Paszkowski-Rogacz M, Kempermann G (2012). The mammalian adult neurogenesis gene ontology (MANGO) provides a structural framework for published information on genes regulating adult hippocampal neurogenesis. PLoS One.

[18] Kempermann G, Jessberger S, Steiner B, Kronenberg G (2004). Milestones of neuronal development in the adult hippocampus. Trends Neurosci..

[19] Ribeiro, F. F. & Xapelli, S. An overview of adult neurogenesis. *Recent Adv. NGF Relat. Mol. Contin. NGF “Saga”* 77–94 (2021). 10.1007/978-3-030-74046-7_7 34453294

[20] Sierra A (2010). Microglia shape adult hippocampal neurogenesis through apoptosis-coupled phagocytosis. Cell Stem Cell.

[21] Plümpe T (2006). Variability of doublecortin-associated dendrite maturation in adult hippocampal neurogenesis is independent of the regulation of precursor cell proliferation. BMC Neurosci..

[22] Pozniak CD, Pleasure SJ (2006). Genetic control of hippocampal neurogenesis. Genome Biol..

[23] Kempermann G, Chesler EJ, Lu L, Williams RW, Gage FH (2006). Natural variation and genetic covariance in adult hippocampal neurogenesis. Proc. Natl. Acad. Sci..

[24] Barón-Mendoza I (2021). Changes in the number and morphology of dendritic spines in the hippocampus and prefrontal cortex of the C58/J mouse model of autism. Front. Cell. Neurosci..

[25] Barón-Mendoza I (2019). Dendritic complexity in prefrontal cortex and hippocampus of the autistic-like mice C58/J. Neurosci. Lett..

[26] Moy SS (2008). Social approach and repetitive behavior in eleven inbred mouse strains. Behav. Brain Res..

[27] Whitehouse CM, Curry-Pochy LS, Shafer R, Rudy J, Lewis MH (2017). Reversal learning in C58 mice: Modeling higher order repetitive behavior. Behav. Brain Res..

[28] The Jackson Laboratory. *Mouse Phenome Database web resource [RRID:SCR_003212]*. https://phenome.jax.org (2023).

[29] Wellcome Trust Sanger Institute. Sanger SNP and indel data, 89+ million locations, 37 inbred strains of mice. MPD:Sanger4. in *Mouse Phenome Database web resource (RRID:SCR_003212), The Jackson Laboratory, Bar Harbor, Maine USA* https://phenome.jax.org (2017).

[30] Bogue MA (2023). Mouse Phenome Database: Towards a more FAIR-compliant and TRUST-worthy data repository and tool suite for phenotypes and genotypes. Nucleic Acids Res..

[31] Adzhubei IA (2010). A method and server for predicting damaging missense mutations. Nat. Methods.

[32] Cembrowski MS, Wang L, Sugino K, Shields BC, Spruston N (2016). Hipposeq: A comprehensive RNA-seq database of gene expression in hippocampal principal neurons. Elife.

[33] Artegiani B (2017). A single-cell RNA sequencing study reveals cellular and molecular dynamics of the hippocampal neurogenic niche. Cell Rep..

[34] Raudvere U (2019). g: Profiler: A web server for functional enrichment analysis and conversions of gene lists (2019 update). Nucleic Acids Res..

[35] Matys V (2006). TRANSFAC® and its module TRANSCompel®: Transcriptional gene regulation in eukaryotes. Nucleic Acids Res..

[36] Reimand J (2016). g: Profiler—a web server for functional interpretation of gene lists (2016 update). Nucleic Acids Res..

[37] Simons Foundation Autism Research Initiative. SFARI Gene. *Database Human gene* https://gene.sfari.org/database/human-gene/ (2023).

[38] Schaaf CP (2020). A framework for an evidence-based gene list relevant to autism spectrum disorder. Nat. Rev. Genet..

[39] Kim H (2022). Dormant state of quiescent neural stem cells links Shank3 mutation to autism development. Mol. Psychiatry.

[40] Liu C (2022). Implication of hippocampal neurogenesis in autism spectrum disorder: Pathogenesis and therapeutic implications. Curr. Neuropharmacol..

[41] Packer A (2016). Neocortical neurogenesis and the etiology of autism spectrum disorder. Neurosci. Biobehav. Rev..

[42] Willsey HR (2021). Parallel in vivo analysis of large-effect autism genes implicates cortical neurogenesis and estrogen in risk and resilience. Neuron.

[43] Cavallucci V, Fidaleo M, Pani G (2016). Neural stem cells and nutrients: Poised between quiescence and exhaustion. Trends Endocrinol. Metab..

[44] Aimone JB (2014). Regulation and function of adult neurogenesis: from genes to cognition. Physiol. Rev..

[45] Zhao C, Deng W, Gage FH (2008). Mechanisms and functional implications of adult neurogenesis. Cell.

[46] Cooper-Kuhn CM (2002). Impaired adult neurogenesis in mice lacking the transcription factor E2F1. Mol. Cell. Neurosci..

[47] Yin B-K, Lázaro D, Wang Z-Q (2023). TRRAP-mediated acetylation on Sp1 regulates adult neurogenesis. Comput. Struct. Biotechnol. J..

[48] Wu Q, Li Y, Xiao B (2013). DISC1-related signaling pathways in adult neurogenesis of the hippocampus. Gene.

[49] Lian G, Chenn A, Ekuta V, Kanaujia S, Sheen V (2019). Formin 2 regulates lysosomal degradation of Wnt-associated β-catenin in neural progenitors. Cereb. Cortex.

[50] Aguilar-Arredondo A, Zepeda A (2018). Memory retrieval-induced activation of adult-born neurons generated in response to damage to the dentate gyrus. Brain Struct. Funct..

[51] Namba T (2011). NMDA receptor regulates migration of newly generated neurons in the adult hippocampus via disrupted-in-schizophrenia 1 (DISC1). J. Neurochem..

[52] Enomoto A (2009). Roles of disrupted-in-schizophrenia 1-interacting protein girdin in postnatal development of the dentate gyrus. Neuron.

[53] Meyer KD, Morris JA (2009). Disc1 regulates granule cell migration in the developing hippocampus. Hum. Mol. Genet..

[54] Gallitano AL, Satvat E, Gil M, Marrone DF (2016). Distinct dendritic morphology across the blades of the rodent dentate gyrus. Synapse.

[55] Gómez-Correa G, Zepeda A (2020). Chronic bumetanide infusion alters young neuron morphology in the dentate gyrus without affecting contextual fear memory. Front. Neurosci..

[56] Jinno S (2011). Topographic differences in adult neurogenesis in the mouse hippocampus: A stereology-based study using endogenous markers. Hippocampus.

[57] Bicker F, Nardi L, Maier J, Vasic V, Schmeisser MJ (2021). Criss-crossing autism spectrum disorder and adult neurogenesis. J. Neurochem..

[58] Stephenson DT (2011). Histopathologic characterization of the BTBR mouse model of autistic-like behavior reveals selective changes in neurodevelopmental proteins and adult hippocampal neurogenesis. Mol. Autism..

[59] Juliandi B (2015). Reduced adult hippocampal neurogenesis and cognitive impairments following prenatal treatment of the antiepileptic drug valproic acid. Stem Cell Rep..

[60] Meng H (2023). The expansion of newborn neurons in hippocampus improves social recognition deficit in a mouse model of autism. Front. Psychiatry.

[61] Du Sert NP (2020). Reporting animal research: Explanation and elaboration for the ARRIVE guidelines 2.0. PLoS Biol..

[62] Paxinos G, Franklin KBJ (2004). The Mouse Brain in Stereotaxic Coordinates.

[63] Schindelin J (2012). Fiji: An open-source platform for biological-image analysis. Nat. Methods.

[64] Keuker JIH, Vollmann-Honsdorf GK, Fuchs E (2001). How to use the optical fractionator: An example based on the estimation of neurons in the hippocampal CA1 and CA3 regions of tree shrews. Brain Res. Protoc..

[65] Mandarim-de-Lacerda CA (2003). Stereological tools in biomedical research. An. Acad. Bras. Cienc..

[66] Bogue MA (2020). Mouse phenome database: A data repository and analysis suite for curated primary mouse phenotype data. Nucleic Acids Res..

