# Supplementary material for: Altered hippocampal neurogenesis in a mouse model of autism revealed by genetic polymorphisms and by atypical development of newborn neurons
Source: Sci Rep. 2024 Feb 26;14:4608. doi: 10.1038/s41598-024-53614-y (PMC10897317; doi:10.1038/s41598-024-53614-y)
Supplement: Supplementary file 1 — Supplementary Information. [file 41598_2024_53614_MOESM1_ESM.docx]

**Supplementary Figures.**


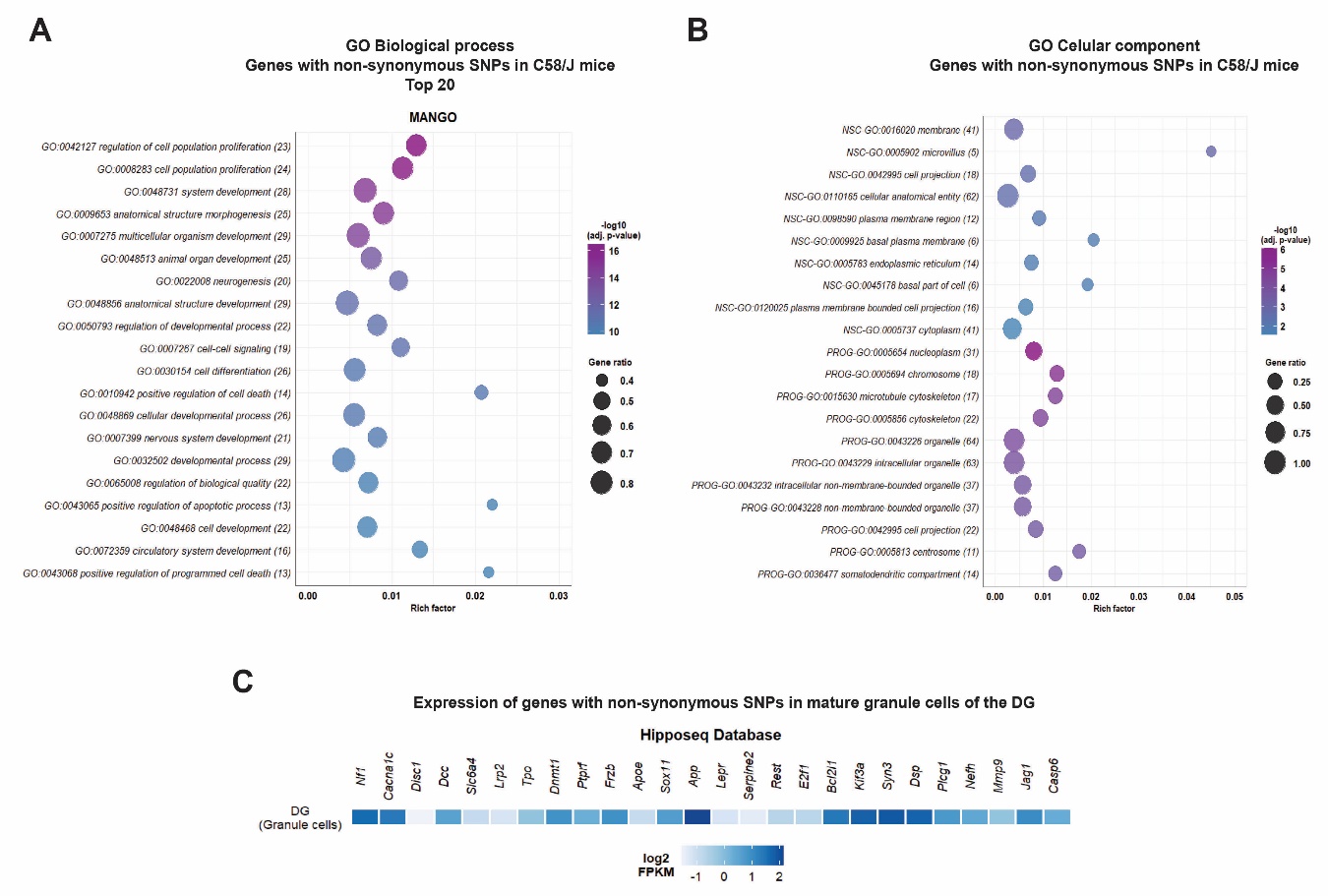


**Supplementary Figure S1. Gene Ontology (GO) enrichment analysis for neurogenesis-associated genes with coding non-synonymous SNPs in the C58/J strain. A)** Top 20 enriched GO terms resulting from the enrichment analysis of the 33 genes with Cn SNPs associated with adult neurogenesis in C58/J mice, according to the MANGO database, in the *biological process* category. **B)** Enriched GO terms resulting from the analysis of the 64 and 78 genes with Cn SNPs associated with NSC and neural progenitors (PROG) in C58/J mice, according to the Artegiani database, in the *cellular component* category. Adjusted p-values were obtained through g:SCS multiple testing correction method on g:Profiler. The number of genes found in each GO term is indicated in parentheses. *Gene ratio:* the number of requested genes found in the functional category divided by the number of genes from the background genome. *Rich factor:* the number of requested genes found in the functional category divided by the number of total genes comprised in the specific functional category. **C)** Expression pattern of the 33 genes with Cn SNPs associated with adult neurogenesis in C58/J mice in mature granule cells (GC) of the mouse DG, according to the Hipposeq platform. Gene expression is represented as log2 FPKM (fragments per kilobase of transcript per million reads mapped).


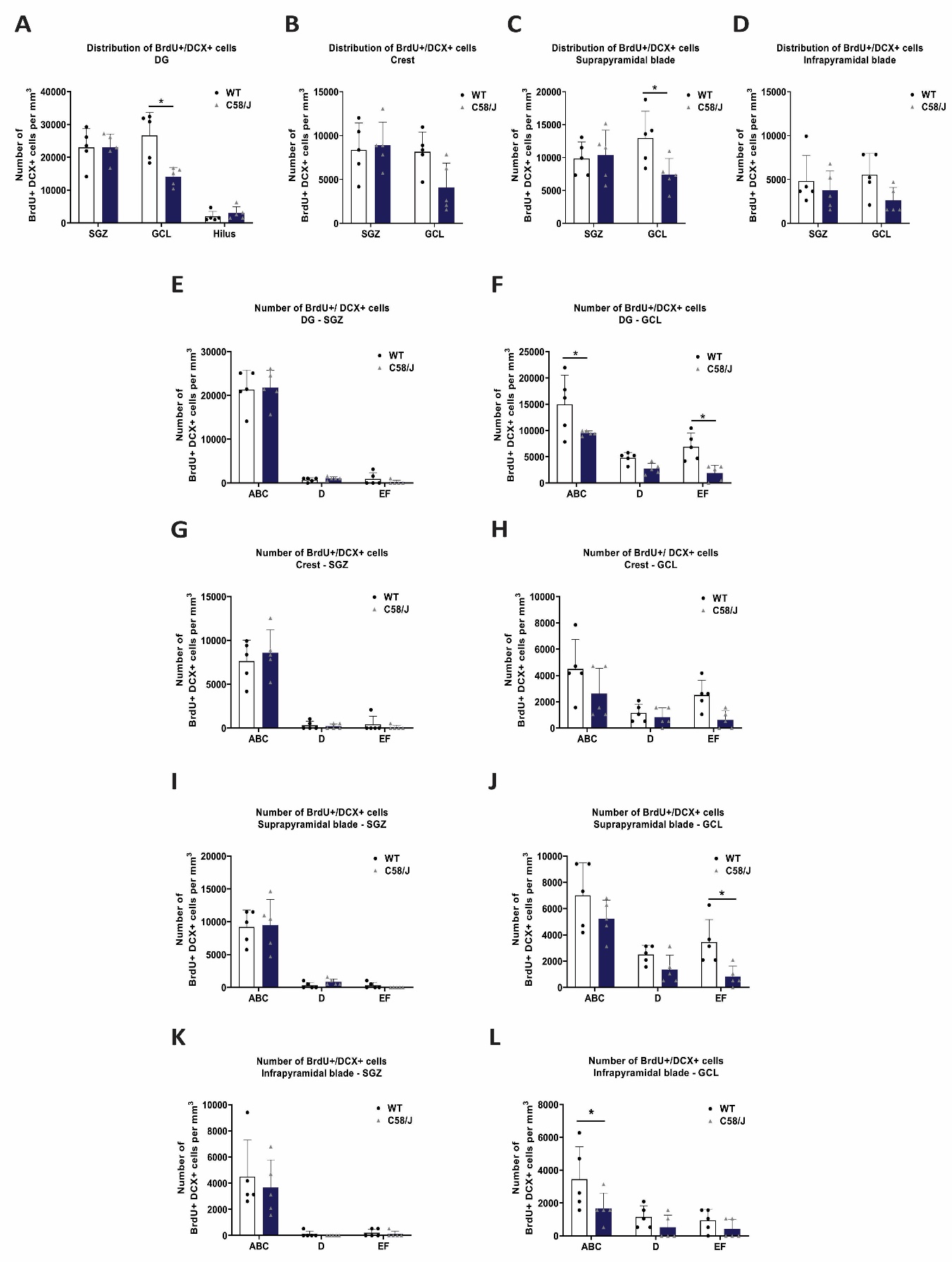


**Supplementary Figure S2. Analysis of the number and morphology of BrdU+/DCX+ cells in the C58/J and WT strains** **according to their distribution in DG regions and layers.** Estimated total number of BrdU+/DCX+ cells distributed within the subgranular zone (SGZ), the granule cell layer (GCL), and the hilus of the whole DG **(A)**, and within the SGZ and GCL of the crest **(B)**, suprapyramidal **(C)**, and infrapyramidal **(D)** blades from both strains. Two-way ANOVA followed by Sidak’s correction: **A) *GCL:*** F_(1, 24)_=5.933, *p=0.0003; **C) *GCL:*** F_(1, 16)_ =2.902, ***p=0.0339.

Estimated total number of BrdU+/DCX+ cells classified according to their morphology within the subgranular zone (SGZ) and the granule cell layer (GCL) of the whole DG **(E, F)**, the crest **(G, H)**, suprapyramidal **(I, J)**, and infrapyramidal **(K, L)** blades from both strains. Two-way ANOVA followed by Sidak’s correction: **F) *GCL-ABC***: F_(1, 24)_=18.53, ***p=0.0106; ***GCL-EF*:** ***p=0.0193; **J) *GCL-EF*:** F_(1, 24)_=11.37, ***p=0.0327; **L) *GCL-ABC*:** F_(1, 24)_=6.506, ***p=0.0386.

All results are expressed as mean ± SD. n =5 animals per group.

**Supplementary Table Legends.**

**Supplementary Table S1.** Definitions of processes and cell stages associated with adult neurogenesis according to the MANGO database.

**Supplementary Table S2.** Information on neurogenesis-associated genes with Cn SNPs in C58/J mice according to the MANGO, NSC & neural progenitors, and Radial Glial Cells datasets.

**Supplementary Table S3.** Impact predictions of Cn SNPs on protein structures as determined by the PolyPhen-2 platform.

**Supplementary Table S4.** Gene Ontology (GO) enrichment analysis of genes with Cn SNPs in C58/J mice reported by the MANGO and NSC & neural progenitors’ datasets.

**Supplementary Table S5.** Genes with Cn SNPs involved in adult neurogenesis in C58/J mice are orthologous to human genes associated with autism spectrum disorder.

**Supplementary Table S6.** Mean group coefficient of error (CE) calculated for the sampling scheme.
