# Supplementary material for: Altered hippocampal neurogenesis in a mouse model of autism revealed by genetic polymorphisms and by atypical development of newborn neurons
Source: Sci Rep. 2024 Feb 26;14:4608. doi: 10.1038/s41598-024-53614-y (PMC10897317; doi:10.1038/s41598-024-53614-y)
Supplement: Supplementary file 2 — Supplementary Table S1. [file 41598_2024_53614_MOESM2_ESM.docx]

**Supplementary Table S1.** Definitions of processes and cell stages associated with adult neurogenesis according to the MANGO database (Overall et al., 2012).

| **Process** | **Definition** |
| --- | --- |
| Proliferation | Refers to the mitotic activity of precursor cells in the SGZ which can be measured by markers such as a pulse or consecutive detection of BrdU, or other endogenous markers such as Ki67, PCNA, Mcm2, or pH3. It is defined as “the number of presently proliferating cells irrespective of the underlying cause”. |
| Differentiation | It stands for an increased proportion of new neurons among the number of surviving newborn cells, or the increased number of neuronal-determined precursor cells assessed by the expression of DCX, Neurod1 or Prox1. |
| Survival | It refers to the continued existence of a cell in the course of development. This requires the BrdU method and assessment at one early and one late time point after BrdU labeling. In most publications, survival relates to an interval of 3–4 weeks after BrdU injection. |
| Dendritogenesis | Dendrite development that occurs during the phase of DCX expression, beginning with type 2b/3 progenitor cells but mostly occurring postmitotically. |
| Migration | Refers to the translocation of a newborn cell from the place of proliferation to the place of terminal maturation and integration. It is associated with the expression of DCX and PSA-NCAM. |
| Maturation | It is defined as the qualitative events besides dendrite and axon elongation that occur between the initiation of neuronal differentiation and fully functional network integration, including the expression of characteristic ion channels, specifics of synapse formation, etc. |
| Axongenesis | Axon development is associated with the late, calretinin, phase of neurogenesis; as well as the establishment of connectivity in CA3 with the formation of synapses in the characteristic boutons of the mossy fibers. |
| Expression | Information about gene expression in a particular cell type in the context of adult neurogenesis without further assessment of that gene’s role in that cell type and hence its ultimate contribution to adult neurogenesis |

| **Cell stage** | **Definition** |
| --- | --- |
| Precursor | Cell types with the ability to proliferate, marked by BrdU. This definition includes type 0-1 cells with self-renewal activity, and progenitor cell types 2a, 2b and 3. |
| Stem cell (Type 1) | Cell with radial glia-like and astrocytic features, including the expression of radial glial marker proteins and GFAP. It can also be identified based on the expression of GFP under the neural enhancer element of the Nestin gene, low proliferative activity, and their radial morphology. |
| Undetermined progenitor (Type 2a) | The highly proliferative intermediate progenitor that has glial features but lacks the radial morphology of type 1 cells. |
| Determined progenitor (Type 2b) | Shows the first signs of neuronal differentiation, assessed by the expression of DCX. |
| Neuroblast like-cell (Type 3) | Cell with a more rounded nucleus, first signs of neurite extension and low proliferative activity. This cell type lacks the immature precursor cell markers such as Sox2 or nestin. |
| New neuron | Postmitotic cell types. It includes both immature and mature neurons, assessed by the expression of DCX/calretinin, calretinin or calbindin. |
| Immature neuron | Refers to cells that, after leaving the cell cycle, express the neuronal marker NeuN and transiently the calcium-binding protein calretinin. |
| Mature neuron | Granule cells identified by the expression of calbindin, but not calretinin. |
| Doublecortin-positive cell | Cells with DCX expression which potentially correspond to progenitor cells (type-2b or type-3) and immature neurons. However, DCX expression alone without the identification of other markers (calretinin, calbindin), is not able to accurately place a cell into a discrete ontological category. |
