# Supplementary material for: Altered hippocampal neurogenesis in a mouse model of autism revealed by genetic polymorphisms and by atypical development of newborn neurons
Source: Sci Rep. 2024 Feb 26;14:4608. doi: 10.1038/s41598-024-53614-y (PMC10897317; doi:10.1038/s41598-024-53614-y)
Supplement: Supplementary file 3 — Supplementary Table S2. [file 41598_2024_53614_MOESM3_ESM.docx]

**Supplementary Table S2.** Information on neurogenesis-associated genes with Cn SNPs in C58/J mice according to the MANGO, NSC & neural progenitors, and Radial Glial Cells datasets.

**Supplementary Table S2.1. Genes with Cn SNPs in C58/J mice (MANGO database)**

33 genes with coding non-synonymous (Cn) SNPs in C58/J mice in comparison to the C57BL/6J strain, identified in the Sanger4 dataset from the MPD platform ^1,2^, are relevant for neurogenesis-associated processes and cell stages according to the MANGO database ^3^, and are expressed in mature granule cells (GC) as reported by the Hipposeq platform ^4^.

| **Gene symbol**  **MANGO** | **Gene**  **name** | **MANGO**  **Subprocess** | **MANGO**  **Cell stage** | **SNP ID**  **Sanger4** | **Coding non-synonymous variant**  **in Sanger4** | **C57BL/6J**  **reference** | **C58/J**  **variant** | **Human orthologous gene symbol** | **SFARI gene score** | **EAGLE**  **score** | **Expression in mature GC**  **Hipposeq** |
| --- | --- | --- | --- | --- | --- | --- | --- | --- | --- | --- | --- |
| *Dnmt1* | DNA methyltransferase (cytosine-5) 1 | - Proliferation - Differentiation - Survival | - New neuron - Precursor - Stem cell (Type 1) | rs29925856 | Cn:Dnmt1:TS:152 | G | C | *NA* | *NA* | *NA* | *Yes* |
| *Ptprf* | protein tyrosine phosphatase, receptor type, F | - Proliferation - Differentiation - Survival | - Precursor - New neuron | rs32074053 | Cn:Ptprf:VA:1184 | A | G | *NA* | *NA* | *NA* | *Yes* |
| *Frzb* | frizzled-related protein | - Proliferation - Maturation | - Mature neuron - Precursor - New neuron - Stem cell (Type 1) | rs51775269 | Cn:Frzb:AV:321 | G | A | *NA* | *NA* | *NA* | *Yes* |
| *Apoe* | apolipoprotein E | - Differentiation - Maturation - Dendritogenesis | - Precursor - New neuron - Stem cell (Type 1) - Undetermined progenitor (Type 2a) | rs13463654 | Cn:Apoe:ED:163 | C | A | *NA* | *NA* | *NA* | *Yes* |
| ***Disc1*** | **disrupted in schizophrenia 1** | - Maturation - Migration - Dendritogenesis | - New neuron - Immature neuron | rs581046352  rs48490186  rs584815636  rs225072421  rs246148673  rs228470821  rs31943450  rs31943453  rs31944226  rs582451279  rs31944231  rs215748054  rs582972657  rs586828569  rs581081884  rs242395305 | Cn:Disc1:HR:9  Cn:Disc1:QR:12  Cn:Disc1:DN:30  Cn:Disc1:RG:119  Cn:Disc1:AG:129  Cn:Disc1:GW:144  Cn:Disc1:FC:153  Cn:Disc1:KT:176  Cn:Disc1:GV:180  Cn:Disc1:PS:195  Cn:Disc1:AP:199  Cn:Disc1:PS:202  Cn:Disc1:ST:286  Cn:Disc1:SP:289  Cn:Disc1:TK:293  Cn:Disc1:QH:640 | A  A  G  C  C  G  T  A  G  C  G  C  G  T  C  G | G  G  A  G  G  T  G  C  T  T  C  T  C  C  A  T | ***DISC1*** | **2** | *NA* | *Yes* |
| *Tnfrsf1b* | tumor necrosis factor receptor superfamily, member 1b | - Proliferation | - Precursor - New neuron | rs27627696  rs27627670  rs48589646  rs27627654  rs27627652 | Cn:Tnfrsf1b:CY:436  Cn:Tnfrsf1b:FS:360  Cn:Tnfrsf1b:IF:283  Cn:Tnfrsf1b:IT:108  Cn:Tnfrsf1b:TS:102 | C  A  T  A  G | T  G  A  G  C | *NA* | *NA* | *NA* | *NA* |
| *Sox11* | SRY-box containing gene 11 | - Proliferation - Expression | - Precursor - New neuron - Determined progenitor (Type 2b) | rs8255798 | Cn:Sox11:AG:177 | G | C | *NA* | *NA* | *NA* | *Yes* |
| *App* | amyloid beta (A4) precursor protein | - Proliferation | - Precursor - New neuron - Stem cell (Type 1) - Doublecortin immunoreactive cell | rs46617381 | Cn:App:GS:221 | C | T | *NA* | *NA* | *NA* | *Yes* |
| *Lepr* | leptin receptor | - Proliferation | - Precursor - New neuron | rs254610605 | Cn:Lepr:IV:359 | A | G | *NA* | *NA* | *NA* | *Yes* |
| *Serpine2* | serine (or cysteine) peptidase inhibitor, clade E, member 2 | - Proliferation - Differentiation - Survival | - Precursor - New neuron | rs221984653 | Cn:Serpine2:IV:313 | T | C | *NA* | *NA* | *NA* | *Yes* |
| ***Slc6a4*** | **solute carrier family 6 (neurotransmitter transporter, serotonin), member 4** | - Proliferation - Survival | - Precursor - New neuron | rs13481111  rs29413009 | Cn:Slc6a4:GE:39  Cn:Slc6a4:KR:152 | G  A | A  G | ***SLC6A4*** | **3** | *NA* | *Yes* |
| *Egf* | epidermal growth factor | - Proliferation - Differentiation | - Precursor - New neuron | rs51615771  rs51409849  rs30802953  rs30555799 | Cn:Egf:ND:589  Cn:Egf:VL:553  Cn:Egf:LR:455  Cn:Egf:GA:430 | T  C  A  C | C  G  C  G | *NA* | *NA* | *NA* | *NA* |
| *Rest* | RE1-silencing transcription factor | - Differentiation | - Precursor - Stem cell (Type 1) - Undetermined progenitor (Type 2a) - Neuroblast-like cell (Type 3) - Mature neuron | rs29727574  rs33582424 | Cn:Rest:ED:534  Cn:Rest:GS:556 | A  G | C  A | *NA* | *NA* | *NA* | *Yes* |
| *E2f1* | E2F transcription factor 1 | - Proliferation | - Precursor - New neuron | rs259162249 | Cn:E2f1:SP:56 | A | G | *NA* | *NA* | *NA* | *Yes* |
| *Bcl2l1* | BCL2-like 1 | - Proliferation | - Precursor | rs27349860 | Cn:Bcl2l1:SN:106 | C | T | *NA* | *NA* | *NA* | *Yes* |
| *Kif3a* | kinesin family member 3A | - Proliferation | - Precursor - Stem cell (Type 1) - Undetermined progenitor (Type 2a) | rs235295691  rs217189586 | Cn:Kif3a:KR:404  Cn:Kif3a:MT:680 | A  T | G  C | *NA* | *NA* | *NA* | *Yes* |
| *Syn3* | synapsin III | - Proliferation | - Precursor - Immature neuron | rs30199006 | Cn:Syn3:GS:65 | C | T | *NA* | *NA* | *NA* | *Yes* |
| *Vipr2* | vasoactive intestinal peptide receptor 2 | - Survival | - New neuron - Undetermined progenitor (Type 2a) - Mature neuron | rs48838258 | Cn:Vipr2:AP:397 | G | C | *NA* | *NA* | *NA* | *NA* |
| ***Nf1*** | **Neurofibromatosis 1** | - Proliferation | - Precursor | rs13481119 | Cn:Nf1:LV:2128 | C | G | ***NF1*** | **1** | **9.3** | *Yes* |
| ***Lrp2*** | **LDL receptor related protein 2** | - Proliferation | - Precursor | rs13467455  rs27974151  rs46664250  rs27958277  rs49433705  rs27958255 | Cn:Lrp2:PQ:4573  Cn:Lrp2:RH:4134  Cn:Lrp2:QR:3263  Cn:Lrp2:FL:2611  Cn:Lrp2:IV:2590  Cn:Lrp2:MT:2421 | G  C  T  A  T  A | T  T  C  C  C  G | ***LRP2*** | **3** | *NA* | *Yes* |
| *Dsp* | desmoplakin | - Proliferation | - Precursor | rs29528874 | Cn:Dsp:SG:1166 | A | G | *NA* | *NA* | *NA* | *Yes* |
| *Plcg1* | phospholipase C, gamma 1 | - Proliferation | - Precursor | rs27303584 | Cn:Plcg1:RH:243 | G | A | *NA* | *NA* | *NA* | *Yes* |
| ***Tpo*** | **thyroid peroxidase** | - Expression | - New neuron | rs49001965  rs46364260  rs47944447 | Cn:Tpo:KN:773  Cn:Tpo:VI:731  Cn:Tpo:AV:42 | C  C  G | G  T  A | ***TPO*** | **3** | *NA* | *Yes* |
| ***Esr2*** | **estrogen receptor 2 (beta)** | - Expression | - Doublecortin immunoreactive cell | rs48183445 | Cn:Esr2:AT:161 | C | T | ***ESR2*** | **3** | *NA* | *NA* |
| ***Cacna1c*** | **calcium channel, voltage-dependent, L type, alpha 1C subunit** | - Expression | - Precursor - Mature Neuron | rs3675286 | Cn:Cacna1c:AV:2110 | G | A | ***CACNAC1C*** | **1** | *NA* | *Yes* |
| *Cnga1* | cyclic nucleotide gated channel alpha 1 | - Expression | - Precursor | rs52256971 | Cn:Cnga1:ED:138 | C | A | *NA* | *NA* | *NA* | *NA* |
| ***Dcc*** | **deleted in colorectal carcinoma** | - Expression | - New neuron | rs30114406 | Cn:Dcc:PS:1315 | G | A | ***DCC*** | **2** | *NA* | *Yes* |
| *Nefh* | neurofilament, heavy polypeptide | - Expression | - New neuron | rs239690638  rs212425084  rs214134975  rs587249762  rs238419426  rs264560184  rs228331256 | Cn:Nefh:ED:1073  Cn:Nefh:PS:823  Cn:Nefh:ED:771  Cn:Nefh:GA:549  Cn:Nefh:GA:531  Cn:Nefh:RP:516 Cn:Nefh::516  Cn:Nefh:SA:27 | T  G  C  C  C  C  A | A  A  G  G  G  G  C | *NA* | *NA* | *NA* | *Yes* |
| *Mmp2* | matrix metallopeptidase 2 | - Expression | - Immature neuron | rs33447269 | Cn:Mmp2:GD:286 | G | A | *NA* | *NA* | *NA* | *NA* |
| *Mmp9* | matrix metallopeptidase 9 | - Expression | - Immature neuron | rs28262053  rs13475086  rs28262043 | Cn:Mmp9:PA:514  Cn:Mmp9:LP:639  Cn:Mmp9:HP:711 | C  T  A | G  C  C | *NA* | *NA* | *NA* | *Yes* |
| *Jag1* | jagged 1 | - Expression | - Doublecortin immunoreactive cell | rs222206013 | Cn:Jag1:TP:571 | T | G | *NA* | *NA* | *NA* | *Yes* |
| *Casp6* | caspase 6 | - Expression | - Precursor | rs16790997 | Cn:Casp6:MV:160 | A | G | *NA* | *NA* | *NA* | *Yes* |
| *Fut4* | fucosyltransferase 4 | - Expression | - Precursor - Stem cell (Type 1) | rs217283253  rs251970372 | Cn:Fut4:RQ:331  Cn:Fut4:RQ:320 | C  C | T  C | *NA* | *NA* | *NA* | *NA* |

NA: Not available data.

Coding non-synonymous (missense): A sequence variant changes one or more bases resulting in a different amino acid sequence, but where the length is preserved ^2^.

The functional annotation (Cn SNP) reported in the Sanger4 database is from the NCBI Single Nucleotide Polymorphism database (dbSNP) build 142. The reference genome corresponds to the C57BL/6J strain ^1,5^.

**Supplementary Table S2.2. Genes with Cn SNPs in C58/J mice (NSC dataset)**

64 genes with coding non-synonymous (Cn) SNPs in C58/J mice in comparison to the C57BL/6J strain, identified in the Sanger4 dataset from the MPD platform ^1,2^, are associated with neural stem cells (NSC) expression profile reported by Artegiani et al. (2017) ^6^.

| **Gene**  **Symbol** | **Gene**  **name** | **SNP ID**  **Sanger4** | **Coding non-synonymous variant**  **in Sanger4** | **C57BL/6J**  **reference** | **C58/J**  **variant** | **Human orthologous gene symbol** | **SFARI**  **gene score** | **EAGLE**  **score** |
| --- | --- | --- | --- | --- | --- | --- | --- | --- |
| *Pla2g7* | phospholipase A2 group VII | rs13468809 | Cn:Pla2g7:VA:427 | T | C | *NA* | *NA* | *NA* |
| *Acsl3* | acyl-CoA synthetase long-chain family member 3 | rs31403527 | Cn:Acsl3:IV:667 | A | G | *NA* | *NA* | *NA* |
| *Cldn10* | claudin 10 | rs30908438  rs30460320 | Cn:Cldn10:AV:181  Cn:Cldn10:TA:198 | C  A | T  G | *NA* | *NA* | *NA* |
| *Prdx6* | peroxiredoxin 6 | rs13474944 | Cn:Prdx6:AG:100  Cn:Prdx6:AV:100  Cn:Prdx6::124 | G | T | *NA* | *NA* | *NA* |
| *Slco1c1* | solute carrier organic anion transporter family member 1C1 | rs8262238  rs225113228  rs8255565 | Cn:Slco1c1:SN:178  Cn:Slco1c1:ND:310  Cn:Slco1c1:TS:314 | G  A  A | A  G  T | *NA* | *NA* | *NA* |
| *Grin2c* | glutamate ionotropic receptor NMDA type subunit 2C | rs264705988  rs29446224  rs29401067 | Cn:Grin2c:AV:1136  Cn:Grin2c:PH:1098  Cn:Grin2c:PL:970 | G  G  G | A  T  A | *NA* | *NA* | *NA* |
| ***Hsd11b1*** | **hydroxysteroid 11-beta dehydrogenase 1** | rs33136315 | Cn:Hsd11b1:DN:18 | C | T | ***HSD11B1*** | **2** | *NA* |
| *Apoe* | apolipoprotein E | rs13463654 | Cn:Apoe:ED:163 | C | A | *NA* | *NA* | *NA* |
| *F3* | coagulation factor III, tissue factor | rs30253761 | Cn:F3:TI:26 | C | T | *NA* | *NA* | *NA* |
| *Psd2* | phosphatidylserine decarboxylase 2 | rs29673194 | Cn:Psd2:LP:554 | T | C | *NA* | *NA* | *NA* |
| *Slc7a10* | solute carrier family 7 (cationic amino acid transporter, y+ system) | rs256404541 | Cn:Slc7a10:HR:17 | A | G | *NA* | *NA* | *NA* |
| *Slc27a1* | solute carrier family 27 (fatty acid transporter), member 1 | rs33084012  rs8262739 | Cn:Slc27a1:SC:50  Cn:Slc27a1:VL:629 | C  G | G  C | *NA* | *NA* | *NA* |
| *Ldhb* | lactate dehydrogenase B | rs254163743  rs32186149 | Cn:Ldhb:RH:252  Cn:Ldhb:MT:245 | C  A | T  G | *NA* | *NA* | *NA* |
| *Lgi4* | leucine rich repeat LGI family member 4 | rs233418515  rs264798503 | Cn:Lgi4:RH:221  Cn:Lgi4:EK:532 | G  G | A  A | *NA* | *NA* | *NA* |
| *Slc15a2* | solute carrier family 15 member 2 | rs50751634 | Cn:Slc15a2:MT:210 | A | G | *NA* | *NA* | *NA* |
| *Vcam1* | vascular cell adhesion molecule 1 | rs13466303 | Cn:Vcam1:ND:693 | T | C | *NA* | *NA* | *NA* |
| *Slc9a3r1* | SLC9A3 regulator 1 | rs249132651  rs29388238 | Cn:Slc9a3r1:PR:200  Cn:Slc9a3r1:AT:272 | T  G | G  A | *NA* | *NA* | *NA* |
| *Eva1a* | eva-1 homolog A, regulator of programmed cell death | rs239720552 | Cn:Eva1a:PS:4 | C | T | *NA* | *NA* | *NA* |
| *Mgst1* | microsomal glutathione S-transferase 1 | rs242835820  rs254541049  rs32013825 | Cn:Mgst1:RG:34  Cn:Mgst1:GS:64  Cn:Mgst1:GV:56 | A  G  G | G  A  T | *NA* | *NA* | *NA* |
| ***Kcnj10*** | **potassium inwardly-rectifying channel, subfamily J, member 10** | rs46006714 | Cn:Kcnj10:TS:262 | C | G | ***KCNJ10*** | **2** | *NA* |
| *Cyp7b1* | cytochrome P450 family 7 subfamily B member 1 | rs13473324 | Cn:Mast2:WS:36 | T | C | *NA* | *NA* | *NA* |
| ***Ank2*** | **ankyrin 2** | rs30583268 | Cn:Ank2:TI:18 | G | A | ***ANK2*** | **1** | **10.8** |
| *Cdh4* | cadherin 4 | rs33484037 | Cn:Cdh4:RH:22 | G | A | *NA* | *NA* | *NA* |
| *Elovl5* | ELOVL fatty acid elongase 5 | rs261670879  rs30437796 | Cn:Elovl5:VM:222  Cn:Elovl5:DE:264 | G  C | A  A | *NA* | *NA* | *NA* |
| *Fmn2* | formin 2 | rs31892648  rs45838473  rs3696849  rs48398548  rs45844610  rs33800912  rs50169104  rs242409105  rs215184893  rs30549466  rs46222699  rs33800711 | Cn:Fmn2:IT:33  Cn:Fmn2:VM:348  Cn:Fmn2:VA:372  Cn:Fmn2:RP:430  Cn:Fmn2:SP:432  Cn:Fmn2:ED:610  Cn:Fmn2:LP:745  Cn:Fmn2:SP:944  Cn:Fmn2:LP:946  Cn:Fmn2:PL:1088  Cn:Fmn2:LP:1142  Cn:Fmn2:DE:1442 | T  G  T  G  T  G  T  T  T  C  T  C | C  A  C  C  C  T  C  C  C  T  C  A | *NA* | *NA* | *NA* |
| *Lars2* | leucyl-tRNA synthetase 2 | rs36883083  rs29976551  rs36300796 | Cn:Lars2:RG:22  Cn:Lars2:IT:479  Cn:Lars2:DN:863 | A  T  G | G  C  A | *NA* | *NA* | *NA* |
| *Mfn1* | mitofusin 1 | rs13475617 | Cn:Mfn1:VI:720 | G | A | *NA* | *NA* | *NA* |
| *Macf1* | microtubule actin crosslinking factor 1 | rs27553570  rs27553513  rs49884863 | Cn:Macf1:VA:3793  Cn:Macf1:GS:2232  Cn:Macf1:LR:1059 | T  A  C  A  G | A  G  T  C  C | *NA* | *NA* | *NA* |
| *Hmgn3* | high mobility group nucleosomal binding domain 3 | rs13474366  rs13474367 | Cn:Hmgn3:EK:95  Cn:Hmgn3:SP:41 | C  A | T  G | *NA* | *NA* | *NA* |
| *Trps1* | transcriptional repressor GATA binding 1 | rs50580568  rs32398060 | Cn:Trps1:GD:1199  Cn:Trps1:VL:817 | C  C | T  G | *NA* | *NA* | *NA* |
| *Ttll3* | tubulin tyrosine ligase-like family, member 3 | rs254654369 | Cn:Ttll3:GR:896 | G | A | *NA* | *NA* | *NA* |
| *Pnisr* | PNN interacting serine and arginine rich protein | rs13460207 | Cn:Pnisr:RG:476 | C | G | *NA* | *NA* | *NA* |
| *Fam171b* | family with sequence similarity 171 member B | rs29555811 | Cn:Fam171b:GE:62 | G | A | *NA* | *NA* | *NA* |
| *Gpi1* | glucose-6-phosphate isomerase 1 | rs32489703 | Cn:Gpi1:ND:95 | T | C | *NA* | *NA* | *NA* |
| *Zfp459* | zinc finger protein 459 | rs29554455  rs29914682  rs48754609  rs29510690  rs30052030  rs29837219  rs29729293 | Cn:Zfp459:ND:212  Cn:Zfp459:NK:161  Cn:Zfp459:VI:9  Cn:Zfp459:SC:81  Cn:Zfp459:EG:79  Cn:Zfp459:DA:77  Cn:Zfp459:SL:65 | T  G  C  G  T  T  G | C  T  T  C  C  G  A | *NA* | *NA* | *NA* |
| *Lama2* | laminin, alpha 2 | rs29383642 | Cn:Lama2:RW:24 | G | A | *NA* | *NA* | *NA* |
| *Pik3ip1* | phosphoinositide-3-kinase interacting protein 1 | rs26884185  rs26884183 | Cn:Pik3ip1:MV:115  Cn:Pik3ip1:PA:124 | A  C | G  G | *NA* | *NA* | *NA* |
| *Rapgef3* | Rap guanine nucleotide exchange factor 3 | rs32066074  rs32264845 | Cn:Rapgef3:HR:457  Cn:Rapgef3:PS:26 | T  G | C  A | *NA* | *NA* | *NA* |
| *Asrgl1* | asparaginase and isoaspartyl peptidase 1 | rs13483522 | Cn:Asrgl1:AT:4 | C | T | *NA* | *NA* | *NA* |
| *Frzb* | frizzled related protein | rs51775269 | Cn:Frzb:AV:321 | G | A | *NA* | *NA* | *NA* |
| *Atxn7* | ataxin 7 | rs225511968  rs45848077  rs31260866 | Cn:Thoc7:AD:110  Cn:Atxn7:VA:232  Cn:Atxn7:EA:875 | G  T  A | T  C  C | *NA* | *NA* | *NA* |
| *Itm2c* | integral membrane protein 2C | rs3714563 | Cn:Itm2c:GA:246 | G | C | *NA* | *NA* | *NA* |
| *Gaa* | alpha glucosidase | rs26999970  rs26999948  rs13469731 | Cn:Gaa:KE:62  Cn:Gaa:DG:434  Cn:Gaa:MV:777 | A  A  A | G  G  G | *NA* | *NA* | *NA* |
| *Ppargc1a* | peroxisome proliferative activated receptor, gamma, coactivator 1 alpha | rs33152841 | Cn:Ppargc1a:RH:675 | C | T | *NA* | *NA* | *NA* |
| *Rnf215* | ring finger protein 215 | rs29434003 | Cn:Rnf215:GC:243 | G | T | *NA* | *NA* | *NA* |
| *Haus2* | HAUS augmin-like complex, subunit 2 | rs51381381 | Cn:Haus2:QK:30 | C | A | *NA* | *NA* | *NA* |
| *Nphs1* | nephrosis 1, nephrin | rs3142457 | Cn:Nphs1:SP:43 | T | C | *NA* | *NA* | *NA* |
| *Suclg1* | succinate-CoA ligase, GDP-forming, alpha subunit | rs263229981 | Cn:Suclg1:VI:175 | G | A | *NA* | *NA* | *NA* |
| *Cln3* | ceroid lipofuscinosis, neuronal 3, juvenile | rs33089967 | Cn:Cln3:HY:120 NC:Cln3 | G | A | *NA* | *NA* | *NA* |
| *Ptprf* | protein tyrosine phosphatase, receptor type, F | rs32074053 | Cn:Ptprf:VA:1184 | A | G | *NA* | *NA* | *NA* |
| *Lepr* | leptin receptor | rs254610605 | Cn:Lepr:IV:359 | A | G | *NA* | *NA* | *NA* |
| *Rad51d* | RAD51 paralog D | rs28209138  rs219351297 | Cn:Rad51d:VA:212  Cn:Rad51d:RQ:177 | A  C | G  T | *NA* | *NA* | *NA* |
| *Slc38a6* | solute carrier family 38 member 6 | rs13481528 | Cn:Slc38a6:AV:345 | C | T | *NA* | *NA* | *NA* |
| *Snrk* | SNF related kinase | rs33499127 | Cn:Snrk:DH:579 | G | C | *NA* | *NA* | *NA* |
| *Zcchc18* | zinc finger, CCHC domain containing 18 | rs31066946 | Cn:Zcchc18:NS:206 | A | G | *NA* | *NA* | *NA* |
| *Lcmt2* | leucine carboxyl methyltransferase 2 | rs27454847  rs33312290  rs27454842  rs27454841  rs27454840  rs27454839 | Cn:Lcmt2:RC:683  Cn:Lcmt2:LP:558  Cn:Lcmt2:VD:250  Cn:Lcmt2:TK:200  Cn:Lcmt2:DH:173  Cn:Lcmt2:EK:124 | G  A  A  G  C  C | A  G  T  T  G  T | *NA* | *NA* | *NA* |
| *Tmem69* | transmembrane protein 69 | rs239340927 | Cn:Tmem69:GS:27 | C | T | *NA* | *NA* | *NA* |
| *Snrnp70* | small nuclear ribonucleoprotein 70 | rs213097687 | Cn:Snrnp70:TS:204 | G | C | *NA* | *NA* | *NA* |
| ***Rfx3*** | **regulatory factor X, 3** | rs30366372 | Cn:Rfx3:AG:715 | G | C | ***RFX3*** | **1** | **15.95** |
| *Eci2* | enoyl-Coenzyme A delta isomerase 2 | rs13464612 | Cn:Eci2:RQ:135 | C | T | *NA* | *NA* | *NA* |
| *Ncan* | neurocan | rs32944017  rs33045210  rs32907915 | Cn:Ncan:KE:947  Cn:Ncan:AP:587  Cn:Ncan:DE:582 | T  C  G | C  G  T | *NA* | *NA* | *NA* |
| *Tapbpl* | TAP binding protein-like | rs30807042  rs29873392  rs30672494 | Cn:Tapbpl:IV:311  Cn:Tapbpl:NT:285  Cn:Tapbpl:GD:224 | T  T  C | C  G  T | *NA* | *NA* | *NA* |
| *Car5a* | carbonic anhydrase 5a, mitochondrial | rs49216355  rs49624203  rs220789260  rs51378363 | Cn:BC048644:SP:18  Cn:BC048644:SL:56  Cn:BC048644:HR:214  Cn:Car5a:LM:215 | T  C  A  G | C  T  G  T | *NA* | *NA* | *NA* |
| *Agl* | amylo-alpha-1, 6-glucosidase, 4-alpha-glucanotransferase | rs30753206 | Cn:Agl:QK:1025 | G | T | *NA* | *NA* | *NA* |

NA: Not available data.

Coding non-synonymous (missense): A sequence variant changes one or more bases resulting in a different amino acid sequence, but where the length is preserved ^2^.

The functional annotation (Cn SNP) reported in the Sanger4 database is from the NCBI Single Nucleotide Polymorphism database (dbSNP) build 142. The reference genome corresponds to the C57BL/6J strain ^1,5^.

**Supplementary Table S2.3. Genes with Cn SNPs in C58/J mice (Neural progenitors’ dataset)**

78 genes with coding non-synonymous (Cn) SNPs in C58/J mice in comparison to the C57BL/6J strain, according to Sanger4 dataset in the MPD platform ^1,2^, are associated with neural progenitors’ expression profile reported by Artegiani et al. (2017) ^6^.

| **Gene**  **Symbol** | **Gene**  **name** | **SNP ID**  **Sanger4** | **Coding non-synonymous variant**  **in Sanger4** | **C57BL/6J**  **reference** | **C58/J**  **variant** | **Human orthologous gene symbol** | **SFARI**  **gene score** | **EAGLE**  **score** |
| --- | --- | --- | --- | --- | --- | --- | --- | --- |
| *Sox11* | SRY (sex determining region Y)-box 11 | rs8255798 | Cn:Sox11:AG:177 | G | C | *NA* | *NA* | *NA* |
| *Nsg2* | neuron specific gene family member 2 | rs26854047 | Cn:Nsg2:AV:79 | C | T | *NA* | *NA* | *NA* |
| *Map1b* | microtubule-associated protein 1B | rs29631491  rs29828789  rs30095909 | Cn:Map1b:AT:2333  Cn:Map1b:IT:1926  Cn:Map1b:LF:1654 | C  A  G | T  G  A | *NA* | *NA* | *NA* |
| *Igsf8* | immunoglobulin superfamily, member 8 | rs51891302  rs50969854 | Cn:Igsf8:HR:221  Cn:Igsf8:TS:489 | A  A | G  T | *NA* | *NA* | *NA* |
| *Rnf165* | ring finger protein 165 | rs38117271 | Cn:Rnf165:NS:128 | T | C | *NA* | *NA* | *NA* |
| *H1f0* | H1.0 linker histone | rs13462306 | Cn:H1f0:AG:190 | C | G | *NA* | *NA* | *NA* |
| *Mllt3* | myeloid/lymphoid or mixed-lineage leukemia; translocated to, 3 | rs28097387 | Cn:Mllt3:MT:352 | A | G | *NA* | *NA* | *NA* |
| *Dusp14* | dual specificity phosphatase 14 | rs13459132 | Cn:Dusp14:WR:45 | A | G | *NA* | *NA* | *NA* |
| *Ncapd2* | non-SMC condensin I complex, subunit D2 | rs30715265  rs46901660 | Cn:Ncapd2:VI:1256  Cn:Ncapd2:GD:574 | C  C | T  T | *NA* | *NA* | *NA* |
| *Osbpl5* | oxysterol binding protein-like 5 | rs246186414  rs254626854 | Cn:Osbpl5:WR:337  Cn:Osbpl5:TM:252 | A  G | G  A | *NA* | *NA* | *NA* |
| ***Chd3*** | **chromodomain helicase DNA binding protein 3** | rs51730412  rs29429936 | Cn:Chd3:ED:1753  Cn:Chd3:WR:390 | C  A | G  G | ***CHD3*** | **1** | *NA* |
| *Cenpf* | centromere protein F | rs50182059 | Cn:Cenpf:KE:1584 | T | C | *NA* | *NA* | *NA* |
| *Sptbn2* | spectrin beta, non-erythrocytic 2 | rs30898646  rs31276767 | Cn:Sptbn2:AT:839  Cn:Sptbn2:RK:1291 | G  G | A  A | *NA* | *NA* | *NA* |
| *Ppdpf* | pancreatic progenitor cell differentiation and proliferation factor | rs49408558  rs216419095 | Cn:Ppdpf:LP:41  Cn:Ppdpf:LP:114 | T  T | C  C | *NA* | *NA* | *NA* |
| *Slc38a1* | solute carrier family 38, member 1 | rs48171807 | Cn:Slc38a1:MV:230 | T | C | *NA* | *NA* | *NA* |
| *Tmpo* | thymopoietin | rs13460679 | Cn:Tmpo:VI:410 | C | T | *NA* | *NA* | *NA* |
| ***Dcc*** | **deleted in colorectal carcinoma** | rs30114406 | Cn:Dcc:PS:1315 | G | A | ***DCC*** | **2** | *NA* |
| ***Myt1l*** | **myelin transcription factor 1-like** | rs29126736  rs50547523 | Cn:Myt1l:ED:141  Cn:Myt1l:SN:806 | A  G | T  A | ***MYT1L*** | **1** | **20.35** |
| *Frmd4b* | FERM domain containing 4B | rs13473708 | Cn:Frmd4b:SN:932 | C | T | *NA* | *NA* | *NA* |
| *Cep170* | centrosomal protein 170 | rs3706759 | Cn:Cep170:MV:164 | T | C | *NA* | *NA* | *NA* |
| *Kif5a* | kinesin family member 5A | rs33857632 | Cn:Kif5a:AT:355 | C | T | *NA* | *NA* | *NA* |
| *Trak1* | trafficking protein, kinesin binding 1 | rs30524308 | Cn:Trak1:RC:10 | C | T | *NA* | *NA* | *NA* |
| ***Pola2*** | **polymerase (DNA directed), alpha 2** | rs30615471 | Cn:Pola2:SP:419 | A | G | ***POLA2*** | **2** | *NA* |
| *Rpl14* | ribosomal protein L14 | rs30384543 | Cn:Rpl14:AV:182 | C | T | *NA* | *NA* | *NA* |
| *Flrt1* | fibronectin leucine rich transmembrane protein 1 | rs242513910 | Cn:Flrt1:RK:344 | C | T | *NA* | *NA* | *NA* |
| *Tpx2* | TPX2, microtubule-associated | rs50152624  rs27349676 | Cn:Tpx2:EK:453  Cn:Tpx2:VL:509 | G  G | A  T | *NA* | *NA* | *NA* |
| *9330159f19rik* | RIKEN cDNA 9330159F19 gene | rs261013725  rs233732096  rs252818487  rs29352185  rs29324855  rs29332276 | Cn:9330159F19Rik:HN:298  Cn:9330159F19Rik:HR:298  Cn:9330159F19Rik:HR:306  Cn:9330159F19Rik:VG:488  Cn:9330159F19Rik:EA:513  Cn:9330159F19Rik:DG:519 | C  A  A  T  A  A | A  G  G  G  C  G | *NA* | *NA* | *NA* |
| ***Atp2b2*** | **ATPase, Ca++ transporting, plasma membrane 2** | rs30756873 | Cn:Atp2b2:ED:731 | C | A | ***ATP2B2*** | **2** | *NA* |
| *Shmt2* | serine hydroxymethyltransferase 2 (mitochondrial) | rs47467180 | Cn:Shmt2:AT:37 | C | T | *NA* | *NA* | *NA* |
| *Serping1* | serine (or cysteine) peptidase inhibitor, clade G, member 1 | rs8273610 | Cn:Serping1:CR:291 | A | G | *NA* | *NA* | *NA* |
| *Sorbs2* | sorbin and SH3 domain containing 2 | rs234307552 | Cn:Sorbs2:VL:147 | G | C | *NA* | *NA* | *NA* |
| *Lig1* | ligase I, DNA, ATP-dependent | rs213219065 | Cn:Lig1:ND:905 | A | G | *NA* | *NA* | *NA* |
| *Mfng* | MFNG O-fucosylpeptide 3-beta-N-acetylglucosaminyltransferase | rs218212244 | Cn:Mfng:QH:248 | C | A | *NA* | *NA* | *NA* |
| *Dctn3* | dynactin 3 | rs13459075 | Cn:Dctn3:AS:106 | C | A | *NA* | *NA* | *NA* |
| *Rpl36* | ribosomal protein L36 | rs33453501 | Cn:Rpl36:FS:7 | T | C | *NA* | *NA* | *NA* |
| *Dact1* | dishevelled-binding antagonist of beta-catenin 1 | rs29132489  rs49637448  rs29222974 | Cn:Dact1:DN:297  Cn:Dact1:AT:585  Cn:Dact1:RP:685 | G  G  G | A  A  C | *NA* | *NA* | *NA* |
| ***Phip*** | **pleckstrin homology domain interacting protein** | rs49517043 | Cn:Phip:TP:1746 | T | G | ***PHIP*** | ***1S*** | *NA* |
| *Bod1* | biorientation of chromosomes in cell division 1 | rs13464522 | Cn:Bod1:SA:17 | A | C | *NA* | *NA* | *NA* |
| *Dgkz* | diacylglycerol kinase zeta | rs32939146  rs32923350  rs13476637 | Cn:Dgkz:SA:196  Cn:Dgkz:PS:157  Cn:Dgkz:RG:143 | A  G  G | C  A  C | *NA* | *NA* | *NA* |
| *Ier2* | immediate early response 2 | rs32599097  rs46425847 | Cn:Ier2:GS:130  Cn:Ier2:VL:46 | C  C | T  G | *NA* | *NA* | *NA* |
| ***Akap9*** | **A kinase (PRKA) anchor protein (yotiao) 9** | rs31188677  rs31180829  rs33433438  rs29732169  rs31168129  rs29563769 | Cn:Akap9:PA:77  Cn:Akap9:AT:1581  Cn:Akap9:VA:2039  Cn:Akap9:KE:2429  Cn:Akap9:GE:3109  Cn:Akap9:QE:3693 | C  G  T  A  G  C | G  A  C  G  A  G | ***AKAP9*** | **2** | *NA* |
| *Brd8* | bromodomain containing 8 | rs13483295 | Cn:Brd8:DE:497 | G | T | *NA* | *NA* | *NA* |
| *Mkrn1* | makorin, ring finger protein, 1 | rs30899669 | Cn:Mkrn1:NY:282 | T | A | *NA* | *NA* | *NA* |
| *Apc* | APC, WNT signaling pathway regulator | rs51271694 | Cn:Apc:RL:1895 | G | T | *NA* | *NA* | *NA* |
| *Ckap2l* | cytoskeleton associated protein 2-like | rs27414803  rs27414792  rs27414791  rs27462801  rs27462800  rs13471578  rs13471576  rs13471577 | Cn:Ckap2l:VA:678  Cn:Ckap2l:HP:660  Cn:Ckap2l:RQ:651  Cn:Ckap2l:MV:293  Cn:Ckap2l:TA:250  Cn:Ckap2l:IV:243  Cn:Ckap2l:HY:168  Cn:Ckap2l:FS:128 | A  T  C  T  T  T  G  A | G  G  T  C  C  C  A  G | *NA* | *NA* | *NA* |
| *Ewsr1* | Ewing sarcoma breakpoint region 1 | rs26916861 | Cn:Ewsr1:TA:97 | T | C | *NA* | *NA* | *NA* |
| *Syne2* | spectrin repeat containing, nuclear envelope 2 | rs29190035  rs51113632  rs29182228  rs48361560  rs47530195  rs49258404  rs49723840  rs46100745  rs225018970  rs29188142  rs46545336  rs246876841  rs47975304  rs51743451  rs51072474  rs51701751  rs46494633  rs29211141  rs29197901  rs48314071 | Cn:Syne2:ED:989  Cn:Syne2:PL:4011  Cn:Syne2:VI:4454  Cn:Syne2:IV:4637  Cn:Syne2:SG:4656  Cn:Syne2:RS:4815  Cn:Syne2:QR:4818  Cn:Syne2:QK:5253  Cn:Syne2:VI:5261  Cn:Syne2:KE:5502  Cn:Syne2:TA:5640  Cn:Syne2:RL:5676  Cn:Syne2:SG:5683  Cn:Syne2:SG:5707  Cn:Syne2:ND:5757  Cn:Syne2:QL:5864  Cn:Syne2:EK:5876  Cn:Syne2:RC:6366  Cn:Syne2:SN:6492  Cn:Syne2:SN:6636 | A  C  G  A  A  C  A  C  G  A  A  G  A  A  A  A  G  C  G  G | C  T  A  G  G  A  G  A  A  G  G  T  G  G  G  T  A  T  A  A | *NA* | *NA* | *NA* |
| *Myt1* | myelin transcription factor 1 | rs27685396 | Cn:Myt1:RH:7 | G | A | *NA* | *NA* | *NA* |
| *Fam214a* | family with sequence similarity 214 member A | rs46398543  rs30472048  rs29837644 | Cn:Gm33615:YD:62  Cn:Fam214a:KN:617  Cn:Fam214a:QH:658 | A  A  G | C  T  T | *NA* | *NA* | *NA* |
| *E2f1* | E2F transcription factor 1 | rs259162249 | Cn:E2f1:SP:56 | A | G | *NA* | *NA* | *NA* |
| *Synrg* | synergin, gamma | rs28225901  rs28225838 | Cn:Synrg:ED:686  Cn:Synrg:SP:868 | A  T | C  C | *NA* | *NA* | *NA* |
| *Neil3* | nei like DNA glycosylase 3 | rs36322259  rs37082574  rs37214799  rs242105085  rs37270056 | Cn:Neil3:KE:585  Cn:Neil3:VA:325  Cn:Neil3:CR:220  Cn:Neil3:PH:90  Cn:Neil3:LP:46 | T  A  A  G  A | C  G  G  T  G | *NA* | *NA* | *NA* |
| *Paxbp1* | PAX3 and PAX7 binding protein 1 | rs4218760 | Cn:Paxbp1:SP:82 | A | G | *NA* | *NA* | *NA* |
| *Rgs12* | regulator of G-protein signaling 12 | rs32544705  rs32537315 | Cn:Rgs12:RK:536  Cn:Rgs12:LS:477 | G  T | A  C | *NA* | *NA* | *NA* |
| *Fmnl2* | formin-like 2 | rs13476502 | Cn:Fmnl2:MV:158 | A | G | *NA* | *NA* | *NA* |
| ***Rfx3*** | **regulatory factor X, 3 (influences HLA class II expression)** | rs30366372 | Cn:Rfx3:AG:715 | G | C | ***RFX3*** | **1** | **15.95** |
| *Wbp1* | WW domain binding protein 1 | rs222169056  rs30989225 | Cn:Wbp1:RC:241  Cn:Wbp1:YH:181 | G  A | A  G | *NA* | *NA* | *NA* |
| *Chd4* | chromodomain helicase DNA binding protein 4 | rs13463121 | Cn:Chd4:TS:1579 | A | T | *NA* | *NA* | *NA* |
| *Hmmr* | hyaluronan mediated motility receptor (RHAMM) | rs28224066  rs231343279  rs243210925 | Cn:Hmmr:TK:414  Cn:Hmmr:AS:153  Cn:Hmmr:AV:94 | G  C  G | T  A  A | *NA* | *NA* | *NA* |
| ***Hnrnpul2*** | **heterogeneous nuclear ribonucleoprotein U-like 2** | rs108171745 | Cn:Hnrnpul2:EG:175 | A | G | ***HNRNPUL2*** | **2** | *NA* |
| *Lama2* | laminin, alpha 2 | rs29383642 | Cn:Lama2:RW:24 | G | A | *NA* | *NA* | *NA* |
| *Cttn* | cortactin | rs38335552 | Cn:Cttn:IV:412 | T | C | *NA* | *NA* | *NA* |
| ***Phf21a*** | **PHD finger protein 21A** | rs27384937 | Cn:Phf21a:PA:325 | C | G | ***PHF21A*** | **1** | **5.35** |
| *Zfp386* | zinc finger protein 386 (Kruppel-like) | rs49308830  rs3726985  rs47600981  rs50778401 | Cn:Zfp386:IV:71  Cn:Zfp386:ND:148  Cn:Zfp386:IV:267  Cn:Zfp386:RT:369 | A  A  A  G | G  G  G  C | *NA* | *NA* | *NA* |
| *Ibtk* | inhibitor of Bruton agammaglobulinemia tyrosine kinase | rs261097195 | Cn:Ibtk:AV:1189 | G | A | *NA* | *NA* | *NA* |
| *Stard7* | START domain containing 7 | rs33279719 | Cn:Stard7:YH:123 | T | C | *NA* | *NA* | *NA* |
| *Htatsf1* | HIV TAT specific factor 1 | rs29061123 | Cn:Htatsf1:VA:577 | T | C | *NA* | *NA* | *NA* |
| *Slc4a7* | solute carrier family 4, sodium bicarbonate cotransporter, member 7 | rs37090346 | Cn:Slc4a7:QR:5 | A | G | *NA* | *NA* | *NA* |
| *Ehbp1* | EH domain binding protein 1 | rs26866453 | Cn:Ehbp1:DE:990 | A | C | *NA* | *NA* | *NA* |
| ***Rad21*** | **RAD21 cohesin complex componen** | rs31823992 | Cn:Rad21:FS:374 | A | G | ***RAD21*** | **S** | *NA* |
| *Gramd1a* | GRAM domain containing 1A | rs32413694 | Cn:Gramd1a:PS:37 | G | A | *NA* | *NA* | *NA* |
| *Hcfc1r1* | host cell factor C1 regulator 1 (XPO1-dependent) | rs4231345 | Cn:Hcfc1r1:VL:118 | G | T | *NA* | *NA* | *NA* |
| ***Epc2*** | **enhancer of polycomb homolog 2** | rs13476485 | Cn:Epc2:LP:382 | T | C | ***EPC2*** | **2** | *NA* |
| *Zfp106* | zinc finger protein 106 | rs13462685  rs13462687  rs33756473  rs32958968  rs33851912  rs33203351  rs220333621  rs238914415  rs260021262  rs248533495  rs27422311  rs232926677  rs238741875  rs27422299  rs49978163  rs33115140  rs27422297  rs237860409  rs27422295  rs33745012  rs33764134  rs27422293  rs233227059 | Cn:Zfp106:IV:1608  Cn:Zfp106:HN:1513  Cn:Zfp106:SN:1273  Cn:Zfp106:AT:1262  Cn:Zfp106:IN:1257  Cn:Zfp106:IF:1257  Cn:Zfp106:LF:1199  Cn:Zfp106:CS:1197  Cn:Zfp106:TM:1196  Cn:Zfp106:SP:1188  Cn:Zfp106:TS:996  Cn:Zfp106:QK:928  Cn:Zfp106:AT:717  Cn:Zfp106:ND:685  Cn:Zfp106:CR:659  Cn:Zfp106:AT:656  Cn:Zfp106:TM:579  Cn:Zfp106:SN:552  Cn:Zfp106:RL:526  Cn:Zfp106:PS:451  Cn:Zfp106:PA:447  Cn:Zfp106:PL:262  Cn:Zfp106:SG:253 | T  G  C  C  A  T  T  C  G  A  T  G  C  T  A  C  G  C  C  G  G  G  T | C  T  T  T  T  A  G  G  A  G  A  T  T  C  G  T  A  T  A  A  C  A  C | *NA* | *NA* | *NA* |
| *Mga* | MAX gene associated | rs50377107  rs27424525  rs27424524  rs27424477  rs27424443  rs27424427  rs13461401  rs29670714  rs27424425 | Cn:Mga:GE:334  Cn:Mga:SC:873  Cn:Mga:TA:903  Cn:Mga:MI:1873  Cn:Mga:CG:2308  Cn:Mga:AT:2651  Cn:Mga:AT:2799  Cn:Mga:IS:2801  Cn:Mga:SL:2828 | G  C  A  G  T  G  G  T  C | A  G  G  A  G  A  A  G  T | *NA* | *NA* | *NA* |
| *Dlgap4* | DLG associated protein 4 | rs33472112  rs33575235  rs27324237 | Cn:Dlgap4:SG:35  Cn:Dlgap4:LP:39  Cn:Dlgap4:IV:154 | A  T  A | G  C  G | *NA* | *NA* | *NA* |
| *Rtf1* | RTF1, Paf1/RNA polymerase II complex component | rs33739475 | Cn:Rtf1:QP:34 | A | C | *NA* | *NA* | *NA* |
| *Mast2* | microtubule associated serine/threonine kinase 2 | rs49184423 | Cn:Mast2:WS:36 | C | G | *NA* | *NA* | *NA* |

NA: Not available data.

Coding non-synonymous (missense): A sequence variant changes one or more bases resulting in a different amino acid sequence, but where the length is preserved ^2^.

The functional annotation (Cn SNP) reported in the Sanger4 database is from the NCBI Single Nucleotide Polymorphism database (dbSNP) build 142. The reference genome corresponds to the C57BL/6J strain ^1,5^.

**Supplementary Table S2.4. Genes with Cn SNPs in C58/J mice (Radial glial cells dataset)**

Thirty-one genes with coding non-synonymous (Cn) SNPs in C58/J mice in comparison to the C57BL/6J strain, according to Sanger4 dataset in the MPD platform ^1,2^, are orthologous to genes dysregulated in radial glial cells (Pax6+, Vimentin+) from individuals with ASD reported by Kim et al. (2022) ^7^.

| **Neurogenesis-associated gene with Cn SNPs in C58/J mice** | **Gene name** | **Human orthologous gen symbol**  **RGC dataset- ASD prefrontal cortex** |
| --- | --- | --- |
| *Cldn10* | claudin 10 | *CLDN10* |
| *Prdx6* | peroxiredoxin 6 | *PRDX6* |
| *Grin2c* | glutamate ionotropic receptor NMDA type subunit 2C | *GRIN2C* |
| *Hsd11b1* | hydroxysteroid 11-beta dehydrogenase 1 like | *HSD11B1L* |
| *Apoe* | apolipoprotein E | *APOE* |
| *F3* | coagulation factor III, tissue factor | *F3* |
| *Psd2* | pleckstrin and Sec7 domain containing 2 | *PSD2* |
| *Slc7a10* | solute carrier family 7 member 10 | *SLC7A10* |
| *Slc27a1* | solute carrier family 27 member 1 | *SLC27A1* |
| *Ldhb* | lactate dehydrogenase B | *LDHB* |
| *Lgi4* | leucine rich repeat LGI family member 4 | *LGI4* |
| *Slc15a2* | solute carrier family 15 member 2 | *SLC15A2* |
| *Slc9a3r1* | sodium-hydrogen antiporter 3 regulator 1 | *SLC9A3R1* |
| *Mgst1* | microsomal glutathione S-transferase 1 | *MGST1* |
| *Kcnj10* | potassium inwardly rectifying channel subfamily J member 10 | *KCNJ10* |
| *Cdh4* | cadherin 4 | *CDH4* |
| *Elovl5* | elongation of very long chain fatty acids protein 5 | *ELOVL5* |
| *Macf1* | microtubule actin crosslinking factor 1 | *MACF1* |
| *Hmgn3* | high mobility group nucleosomal binding domain 3 | *HMGN3* |
| *Trps1* | transcriptional repressor GATA binding 1 | *TRPS1* |
| *Ttll3* | tubulin tyrosine ligase like 3 | *TTLL3* |
| *Fam171b* | family with sequence similarity 171 member B | *FAM171B* |
| *Slco1c1* | solute carrier organic anion transporter family member 1C1 | *SLCO1C1* |
| *Fmn2* | formin 2 | *FMN2* |
| *Pnisr* | PNN interacting serine and arginine rich protein | *PNISR* |
| *Dnmt1* | DNA methyltransferase 1 | *DNMT1* |
| *Ptprf* | protein tyrosine phosphatase receptor type F | *PTPRF* |
| *App* | amyloid beta precursor protein | *APP* |
| *Serpine2* | serpin family E member 2 | *SERPINE2* |
| *Rest* | RE1 silencing transcription factor | *REST* |
| *Frzb* | frizzled related protein | *FRZB* |

**Supplementary References.**

1. Wellcome Trust Sanger Institute. Sanger SNP and indel data, 89+ million locations, 37 inbred strains of mice. MPD:Sanger4. *Mouse Phenome Database web resource (RRID:SCR_003212), The Jackson Laboratory, Bar Harbor, Maine USA* https://phenome.jax.org (2017).

2. The Jackson Laboratory. Mouse Phenome Database web resource [RRID:SCR_003212]. https://phenome.jax.org (2023).

3. Overall, R. W., Paszkowski-Rogacz, M. & Kempermann, G. The mammalian adult neurogenesis gene ontology (MANGO) provides a structural framework for published information on genes regulating adult hippocampal neurogenesis. *PLoS One* **7**, e48527 (2012).

4. Cembrowski, M. S., Wang, L., Sugino, K., Shields, B. C. & Spruston, N. Hipposeq: a comprehensive RNA-seq database of gene expression in hippocampal principal neurons. *Elife* **5**, e14997 (2016).

5. Keane, T. M. *et al.* Mouse genomic variation and its effect on phenotypes and gene regulation. *Nature* **477**, 289–294 (2011).

6. Artegiani, B. *et al.* A single-cell RNA sequencing study reveals cellular and molecular dynamics of the hippocampal neurogenic niche. *Cell Rep.* **21**, 3271–3284 (2017).

7. Kim, H. *et al.* Dormant state of quiescent neural stem cells links Shank3 mutation to autism development. *Mol. Psychiatry* **27**, 2751–2765 (2022).
