# Supplementary material for: Altered hippocampal neurogenesis in a mouse model of autism revealed by genetic polymorphisms and by atypical development of newborn neurons
Source: Sci Rep. 2024 Feb 26;14:4608. doi: 10.1038/s41598-024-53614-y (PMC10897317; doi:10.1038/s41598-024-53614-y)
Supplement: Supplementary file 4 — Supplementary Table S3. [file 41598_2024_53614_MOESM4_ESM.docx]

**Supplementary Table S3.** Impact predictions of Cn SNPs on protein structures as determined by the PolyPhen-2 platform.

| **Gene symbol** | **Gene**  **name** | **SNP ID**  **Sanger4** | **Coding non-synonymous variant**  **in Sanger4** | **C57BL/6J**  **reference** | **C58/J**  **variant** | **PolyPhen-2**  **Impact prediction** | **Score**  **FPR** | **Sensitivity**  **TPR** | **Specificity**  **TNR** |
| --- | --- | --- | --- | --- | --- | --- | --- | --- | --- |
| *Disc1* | disrupted in schizophrenia 1 | rs581046352  rs48490186  rs584815636  rs225072421  rs246148673  rs228470821  rs31943450  rs31943453  rs31944226  rs582451279  rs31944231  rs215748054  rs582972657  rs586828569  rs581081884  rs242395305 | Cn:Disc1:HR:9  Cn:Disc1:QR:12  Cn:Disc1:DN:30  Cn:Disc1:RG:119  Cn:Disc1:AG:129  Cn:Disc1:GW:144  Cn:Disc1:FC:153  Cn:Disc1:KT:176  Cn:Disc1:GV:180  Cn:Disc1:PS:195  Cn:Disc1:AP:199  Cn:Disc1:PS:202  Cn:Disc1:ST:286  Cn:Disc1:SP:289  Cn:Disc1:TK:293  Cn:Disc1:QH:640 | A  A  G  C  C  G  T  A  G  C  G  C  G  T  C  G | G  G  A  G  G  T  G  C  T  T  C  T  C  C  A  T | NA  NA  NA  BENIGN  BENIGN  BENIGN  BENIGN  POSSIBLY DAMAGING  PROBABLY DAMAGING  BENIGN  BENIGN  PROBABLY DAMAGING  BENIGN  BENIGN  BENIGN  NA | NA  NA  NA  0.000  0.000   0.009  0.000  0.870  0.996  0.207  0.001  0.993  0.401  0.066  0.014  NA | NA  NA  NA  1.00  1.00  0.96  1.00  0.83  0.55  0.92  0.99  0.7  0.9  0.94  0.96  NA | NA  NA  NA  0.00  0.00  0.77  0.00  0.93  0.98  0.88  0.15  0.97  0.9  0.84  0.79  NA |
| *Fmn2* | formin 2 | rs31892648  rs45838473  rs3696849  rs48398548  rs45844610  rs33800912  rs50169104  rs242409105  rs215184893  rs30549466  rs46222699  rs33800711 | Cn:Fmn2:IT:33  Cn:Fmn2:VM:348  Cn:Fmn2:VA:372  Cn:Fmn2:RP:430  Cn:Fmn2:SP:432  Cn:Fmn2:ED:610  Cn:Fmn2:LP:745  Cn:Fmn2:SP:944  Cn:Fmn2:LP:946  Cn:Fmn2:PL:1088  Cn:Fmn2:LP:1142  Cn:Fmn2:DE:1442 | T  G  T  G  T  G  T  T  T  C  T  C | C  A  C  C  C  T  C  C  C  T  C  A | BENIGN  PROBABLY DAMAGING  BENIGN  PROBABLY DAMAGING  BENIGN  BENIGN  BENIGN  NA  NA  NA  BENIGN  POSSIBLY DAMAGING | 0.006  0.989  0.006  0.998  0.005  0.005  0.005  NA  NA  NA  0.019  0.564 | 0.97  0.72  0.97  0.27  0.97  0.97  0.97  NA  NA  NA  0.95  0.88 | 0.75  0.97  0.75  0.99  0.74  0.74  0.74  NA  NA  NA  0.8  0.91 |
| *Zfp106* | zinc finger protein 106 | rs13462685  rs13462687  rs33756473  rs32958968  rs33851912  rs33203351  rs220333621  rs238914415  rs260021262  rs248533495  rs27422311  rs232926677  rs238741875  rs27422299  rs49978163  rs33115140  rs27422297  rs237860409  rs27422295  rs33745012  rs33764134  rs27422293  rs233227059 | Cn:Zfp106:IV:1608  Cn:Zfp106:HN:1513  Cn:Zfp106:SN:1273  Cn:Zfp106:AT:1262  Cn:Zfp106:IN:1257  Cn:Zfp106:IF:1257  Cn:Zfp106:LF:1199  Cn:Zfp106:CS:1197  Cn:Zfp106:TM:1196  Cn:Zfp106:SP:1188  Cn:Zfp106:TS:996  Cn:Zfp106:QK:928  Cn:Zfp106:AT:717  Cn:Zfp106:ND:685  Cn:Zfp106:CR:659  Cn:Zfp106:AT:656  Cn:Zfp106:TM:579  Cn:Zfp106:SN:552  Cn:Zfp106:RL:526  Cn:Zfp106:PS:451  Cn:Zfp106:PA:447  Cn:Zfp106:PL:262  Cn:Zfp106:SG:253 | T  G  C  C  A  T  T  C  G  A  T  G  C  T  A  C  G  C  C  G  G  G  T | C  T  T  T  T  A  G  G  A  G  A  T  T  C  G  T  A  T  A  A  C  A  C | BENIGN  BENIGN  BENIGN  BENIGN  POSSIBLY DAMAGING  POSSIBLY DAMAGING  BENIGN  BENIGN  BENIGN  POSSIBLY DAMAGING  BENIGN  PROBABLY DAMAGING  POSSIBLY DAMAGING  BENIGN  POSSIBLY DAMAGING  BENIGN  POSSIBLY DAMAGING  BENIGN  POSSIBLY DAMAGING  BENIGN  BENIGN  POSSIBLY DAMAGING  BENIGN | 0.013  0.004  0.001  0.000  0.819  0.694  0.000  0.000  0.329  0.597  0.001  0.993  0.845  0.001  0.846  0.001  0.911  0.106  0.917  0.024  0.001  0.500  0.006 | 0.96  0.97  0.99  1.00  0.84  0.86  1.00  1.00  0.90  0.87  0.99  0.7  0.83  0.99  0.83  0.99  0.81  0.93  0.81  0.95  0.99  0.88  0.97 | 0.78  0.59  0.15  0.00  0.93  0.92  0.00  0.00  0.89  0.91  0.15  0.97  0.93  0.15  0.93  0.15  0.94  0.86  0.94  0.81  0.15  0.90  0.75 |

Estimates of the false positive rate (FPR/Score), true positive rate (TPR/Sensitivity), and true negative rate (TNR/Specificity) are provided for each Cn SNP in the *Disc1*, *Fmn2*, and *Zfp106* genes. According to the PolyPhen-2 platform trained by the Humdiv model, the functional significance of an allele replacement (the prediction of its impact on the protein structure) is qualitatively classified based on pairs of false positive rate (FPR) thresholds: *benign* (indicating a low probability of protein damage), *possibly damaging* (indicating a less confident prediction of protein damage), or *probably damaging* (a more confident prediction of protein damage).

**NA: not available score**. The lack of data does not allow for making a prediction of protein impact according to PolyPhen-2.
