# Supplementary material for: Altered hippocampal neurogenesis in a mouse model of autism revealed by genetic polymorphisms and by atypical development of newborn neurons
Source: Sci Rep. 2024 Feb 26;14:4608. doi: 10.1038/s41598-024-53614-y (PMC10897317; doi:10.1038/s41598-024-53614-y)
Supplement: Supplementary file 6 — Supplementary Table S5. [file 41598_2024_53614_MOESM6_ESM.docx]

**Supplementary Table S5.** Genes with Cn SNPs involved in adult neurogenesis in C58/J mice are orthologous to human genes associated with autism spectrum disorder.

| **Gene symbol** | **Gene name** | **Human orthologous gene symbol** | **SFARI gene score** | **EAGLE score** | **Database** |
| --- | --- | --- | --- | --- | --- |
| *Nf1* | Neurofibromatosis 1 | *NF1* | 1 | 9.3 | MANGO |
| *Cacna1c* | calcium channel, voltage-dependent, L type, alpha 1C subunit | *CACNAC1C* | 1 | NA | MANGO |
| *Chd3* | chromodomain helicase DNA binding protein 3 | *CHD3* | 1 | NA | NSC & neural progenitors (Artegiani et al., 2017) |
| *Myt1l* | myelin transcription factor 1-like | *MYT1L* | 1 | 20.35 | NSC & neural progenitors (Artegiani et al., 2017) |
| *Phf21a* | PHD finger protein 21A | *PHF21A* | 1 | 5.35 | NSC & neural progenitors (Artegiani et al., 2017) |
| *Ank2* | ankyrin 2 | *ANK2* | 1 | 10.8 | NSC & neural progenitors (Artegiani et al., 2017) |
| *Rfx3* | regulatory factor X, 3 | *RFX3* | 1 | 15.95 | NSC & neural progenitors (Artegiani et al., 2017) |
| *Phip* | pleckstrin homology domain interacting protein | *PHIP* | 1 | NA | NSC & neural progenitors (Artegiani et al., 2017) |
| *Rad21* | RAD21 cohesin complex componen | *RAD21* | S | NA | NSC & neural progenitors (Artegiani et al., 2017) |
| *Disc1* | disrupted in schizophrenia 1 | *DISC1* | 2 | NA | MANGO |
| *Pola2* | polymerase (DNA directed), alpha 2 | *POLA2* | 2 | NA | NSC & neural progenitors (Artegiani et al., 2017) |
| *Atp2b2* | ATPase, Ca++ transporting, plasma membrane 2 | *ATP2B2* | 2 | NA | NSC & neural progenitors (Artegiani et al., 2017) |
| *Akap9* | A kinase (PRKA) anchor protein (yotiao) 9 | *AKAP9* | 2 | NA | NSC & neural progenitors (Artegiani et al., 2017) |
| *Hnrnpul2* | heterogeneous nuclear ribonucleoprotein U-like 2 | *HNRNPUL2* | 2 | NA | NSC & neural progenitors (Artegiani et al., 2017) |
| *Epc2* | enhancer of polycomb homolog 2 | *EPC2* | 2 | NA | NSC & neural progenitors (Artegiani et al., 2017) |
| *Hsd11b1* | hydroxysteroid 11-beta dehydrogenase 1 | *HSD11B1* | 2 | NA | NSC & neural progenitors (Artegiani et al., 2017) |
| *Kcnj10* | potassium inwardly-rectifying channel, subfamily J, member 10 | *KCNJ10* | 2 | NA | NSC & neural progenitors (Artegiani et al., 2017) |
| *Dcc* | deleted in colorectal carcinoma | *DCC* | 2 | NA | MANGO  NSC & neural progenitors (Artegiani et al., 2017) |
| *Slc6a4* | solute carrier family 6 (neurotransmitter transporter, serotonin), member 4 | *SLC6A4* | 3 | NA | MANGO |
| *Lrp2* | LDL receptor related protein 2 | *LRP2* | 3 | NA | MANGO |
| *Tpo* | thyroid peroxidase | *TPO* | 3 | NA | MANGO |
| *Esr2* | estrogen receptor 2 (beta) | *ESR2* | 3 | NA | MANGO |

(NA) Not available data.
