# Supplementary material for: Altered hippocampal neurogenesis in a mouse model of autism revealed by genetic polymorphisms and by atypical development of newborn neurons
Source: Sci Rep. 2024 Feb 26;14:4608. doi: 10.1038/s41598-024-53614-y (PMC10897317; doi:10.1038/s41598-024-53614-y)
Supplement: Supplementary file 7 — Supplementary Table S6. [file 41598_2024_53614_MOESM7_ESM.docx]

**Supplementary Table S6.** Mean group coefficient of error (CE) calculated for the sampling scheme.

CE should be under 0.10 as indicated by (Keuker et al., 2001).

| **Mean coefficient error (CE)** | | | | | |
| --- | --- | --- | --- | --- | --- |
| **Crest** | | **Suprapyramidal blade** | | **Infrapyramidal blade** | |
| **C57 BL/6J** | **C58/J** | **C57 BL/6J** | **C58/J** | **C57 BL/6J** | **C58/J** |
| 0.070516462 | 0.051519661 | 0.096971676 | 0.057826273 | 0.070365789 | 0.049780338 |
